# HVEM costimulatory domain boosts CAR T cell efficacy against solid tumors via enhanced TRAF-mediated TNF signaling

**DOI:** 10.1186/s12964-025-02648-4

**Published:** 2026-01-08

**Authors:** Shishuo Sun, Wanxin Zhao, Yibing Liang, Ying Xue, Qihong Li, Yifan Yuan, Zhenyu Wang, Xiaoge Gao, Yizhou Yao, Haiheng Xu, Hailong Li, Xiaoxiao Liu, Patrick Ming-Kuen Tang, Junnian Zheng, Qing Zhang

**Affiliations:** 1 Cancer Institute, Cellular Therapeutics School of Medicine, Xuzhou Medical University, Xuzhou, Jiangsu 221004 P.R. China; 2 Center of Clinical Oncology, The Affiliated Hospital of Xuzhou Medical University, Jiangsu Xuzhou, 221002 P.R. China; 3 Jiangsu Center for the Collaboration and Innovation of Cancer Biotherapy, Cancer Institute, Xuzhou Medical University, Xuzhou, Jiangsu P. R. China; 4 Department of General Surgery, The First Affiliated Hospital of Soochow University, Suzhou, Jiangsu P.R. China; 5 Department of Urology, The Affiliated Hospital of Xuzhou Medical University, Xuzhou, Jiangsu P.R. China; 6 Department of Radiation Oncology, Cancer Center, The Affiliated Hospital of Xuzhou Medical University, Xuzhou, Jiangsu P.R. China; 7 Department of Anatomical and Cellular Pathology, State Key Laboratory of Translational Oncology, The Chinese University of Hong Kong, Shatin, Hong Kong P.R. China

**Keywords:** HVEM, TRAF, CAR T, Costimulatory molecules, Solid tumor

## Abstract

**Background:**

Chimeric antigen receptor (CAR) T cell therapy has shown success in hematological malignancies, but its efficacy against solid tumors remains limited. Herpesvirus entry mediator (HVEM), a co-stimulatory molecule, enhances CAR T cell activity, though the underlying mechanisms are unclear. This study investigates how HVEM-based CAR T cells outperform those using 4-1BB in solid tumor models.

**Methods:**

Second-generation CAR T cells targeting carbonic anhydrase IX (CAIX) were engineered with either 4-1BB, wild type (WT) HVEM, or AVEE mutation (MUT) HVEM co-stimulatory domains. In vitro cytotoxicity, cytokine release, and signaling pathways were assessed using real-time cellular analysis, ELISA, and Western blotting. RNA sequencing and gene set enrichment analysis (GSEA) compared transcriptional profiles. Co-immunoprecipitation and mass spectrometry identified TNF receptor-associated factor (TRAF) protein interactions. In vivo efficacy was evaluated in orthotopic and subcutaneous renal cancer models using NPG mice. Metabolic activity was measured via Seahorse assays.

**Results:**

HVEM-CAR T cells exhibited stronger cytotoxicity, cytokine release, and proliferation than 4-1BB-CAR T cells in response to target cancer cell stimulation in vitro. RNA sequencing and Western blotting revealed enhanced TNF signaling in HVEM-CAR T cells, with higher phosphorylation of AKT and ERK1/2, accompanied by elevated metabolic levels. HVEM recruited TRAF1, TRAF2, TRAF3, and TRAF5 via its AVEE motif, whereas 4-1BB only bound TRAF1-3. Mutation of the AVEE motif disrupted TRAF binding, reduced signaling activation, and impaired metabolic and antitumor functions. In vivo, HVEM-CAR T cells showed superior tumor control, prolonged survival, and increased intratumoral abundance compared to 4-1BB-CAR or AVEE-mutated HVEM-CAR T cells.

**Conclusions:**

HVEM enhances CAR T cell efficacy against solid tumors by robustly activating TNF signaling through TRAF recruitment, particularly via the AVEE motif. These findings highlight HVEM as a promising co-stimulatory domain for improving CAR T cell therapy in solid tumors, with implications for metabolic reprogramming and sustained antitumor activity.

**Supplementary Information:**

The online version contains supplementary material available at 10.1186/s12964-025-02648-4.

## Background

Chimeric antigen receptor-engineered T (CAR T) cell therapy has achieved significant success in treating hematological malignancies [1]. Currently, six CAR T cell products based on the second-generation CAR structure have been approved by the US Food and Drug Administration (FDA) and seven by the Chinese National Medical Products Administration for 12 indications of hematological cancers [2, 3]. A second-generation CAR consists of an extracellular ligand-binding domain, most commonly a single-chain variable fragment (scFv), a spacer domain, a transmembrane domain, one co-stimulatory domain (CSD), and an intracellular domain derived from CD3ζ [4]. The intracellular domains of 4-1BB and CD28 serve as CSDs in the CAR structure for all approved products [5, 6]. However, the efficacy of CAR T cells utilizing 4-1BB or CD28 CSDs against solid tumors is quite limited [7]. Therefore, identifying more effective co-stimulatory molecules may be one way to enhance the efficacy of CAR T therapy for solid tumors.

The herpes virus entry mediator (HVEM), also known as CD270, is a co-stimulatory molecule belonging to the tumor necrosis factor receptor superfamily (TNFRSF) [8]. Our previous research has demonstrated that HVEM-based CAR T cells exhibit superior efficacy against solid tumors in renal and colon cancer models compared to 4-1BB- or CD28-based CAR T cells, attributed to enhanced CAR T cell metabolic activities [9]. However, the underlying molecular mechanisms remain unclear. Previous studies, including our own, have shown that the efficacy of CAR T cells using 4-1BB and CD28 CSDs against solid tumors is comparable [9, 10]. Since CD28 belongs to the IgG superfamily, and 4-1BB and HVEM are members of the TNFRSF, the molecular mechanisms by which these two families of co-stimulatory molecules regulate CAR T cells differ [11]. Therefore, this study primarily aims to elucidate the molecular mechanisms through which HVEM CSD enhances CAR T cell metabolic activity and therapeutic efficacy against solid tumors, comparing it with 4-1BB CSD.

The current study demonstrates that, compared to 4-1BB-CAR T cells, HVEM-CAR T cells strongly activate the TNF signaling pathway, particularly by phosphorylating AKT and ERK1/2 in response to tumor antigen stimulation, which confers higher metabolic levels and, as a result, more potent anti-tumor effector functions. Mechanistically, while 4-1BB-CAR T cells recruit TRAF1, TRAF2, and TRAF3, HVEM-CAR T cells recruit a greater number of TRAF1, TRAF2, and TRAF3 molecules and additionally bind TRAF5 through the AVEE motif. Mutation of the AVEE motif significantly inhibited the association between HVEM-CAR T cells and TRAFs, reduced the levels of phosphorylated AKT and ERK1/2, and decreased the metabolic activities of HVEM-CAR T cells, thereby diminishing the antitumor efficacy of HVEM-CAR T cells. Thus, this study reveals the molecular mechanism by which HVEM CSD promotes CAR T cell antitumor efficacy, providing evidence for the clinical application of HVEM-CAR T cells against solid tumors.

## Methods

### Materials and reagents

The X-VIVO 15 medium was obtained from Lonza (Switzerland) and RPMI-1640 and DMEM were purchased from KeyGEN BioTECH (Nanjing, China). Penicillin/streptomycin solution and fetal bovine serum (FBS) were bought from BioChannel Biotechnology Co., Ltd (Nanjing, China). In addition, Dynabeads™ Human T-Activator CD3/CD28 was obtained from Gibco (11132D), recombinant human IL-2 (rhIL-2) was purchased from PrimeGene (Shanghai, China), and recombinant human carbonic anhydrase 9 (rhCAIX) was obtained from MedChemExpress (Shanghai, China). Information regarding antibodies for Western blotting and flow cytometry are provided in supplementary Tables 1 and 2.

### Cell culture

Human acute T cell leukemia cell line Jurkat (clone E6-1) was donated from BioChannel Biotechnology (Nanjing, China), human renal cancer cell line OSRC-2 was purchased from Shanghai Tongwei Biotechnology Co., LTD., and human renal cancer cell line Ketr-3 was bought from National Collection of Authenticated Cell Cultures (Shanghai, China). OSRC-2 cells were transfected using a lentivirus with luciferase expression, and puromycin was used to select the luciferase-overexpressing cells OSRC-2^Luc+^. HVEM-CAR Jurkat cell lines with TRAF deficiency were established using lentivirus expressing specific shRNA and selected via puromycin. The TRAF-targeting shRNA sequences are listed in supplementary Table 3. Jurkat and OSRC-2 cells were cultured in RPMI-1640 medium, supplemented with 10% FBS and 1% penicillin/streptomycin; Ketr-3 cells were cultured in DMEM, supplemented with 10% FBS and 1% penicillin/streptomycin; and T cells were cultured in X-VIVO 15 medium, supplemented with 10% FBS, 1% penicillin/streptomycin, and 200 IU/mL rhIL-2. All cells were cultured at 37℃ in a 5% CO_2_ humidified atmosphere. Cell line authentication and *Mycoplasma* testing were performed by the vendor.

### CAR plasmid construction and lentiviral packaging

Second-generation CARs were constructed by fusing a myc tag, an anti-human CAIX scFv with the hinge and transmembrane domains of human CD8α, and the intracellular regions of a CSD (4-1BB or HVEM) and CD3ζ. These constructs were cloned into the lentiviral vector plasmid pRRL. Plasmid harboring the AVEE mutation, which was modified to AVAA in HVEM-CAR was obtained using overlap PCR with site-directed mutagenesis primers.

The lentiviral particles were generated by transfecting HEK 293T cells with the lentiviral vector plasmid pRRL encoding CAIX-CAR with various CSDs along with the Δ8.9 packaging plasmid and envelope plasmid VSVG using PEI. Culture supernatants containing lentiviral particles were harvested at 48 h after plasmid transfection. Virus titration was performed via flow cytometry.

### CAR T cell preparation

Human peripheral blood mononuclear cells (PBMCs) were isolated using Lymphoprep gradient (07851/07861, StemCell Technologies) from peripheral blood donated by healthy donors and stored in liquid nitrogen for CAR T preparation. PBMCs were eventually thawed and activated with Human T-Activator CD3/CD28 Dyna-beads at a ratio of 1:1, and 24 h later, PBMCs were infected with the lentivirus encoding various CAR genes.

### Real-time cellular analysis (RTCA)

The cytotoxicity of CAR T cells in vitro was determined using the Agilent xCELLigence RTCA instrument according to a protocol described earlier [12, 13]. In brief, the background impedance was measured by adding 50 µL of the cell culture medium to an E-plate (ACEA Biosciences). OSRC-2 or Ketr-3 cancer cells were seeded into the E-plate at a concentration of 3,000 cells/well or 5,000 cells/well, respectively, and allowed to adhere before CAR T cell addition at the indicated effector cell: target cancer cell (E: T) ratios. A target cancer cell only controls indicated cancer cell proliferation. The CAR T cytotoxic activity was determined based on attached viable target cells (cell index value). Data are shown after normalizing the cell index with the time point of CAR T cell addition (normalized cell index).

### Enzyme-linked immunosorbent assay (ELISA)

CAR T cells were cocultured with the target cancer cells for 24 h at the indicated E: T ratios. Media from the cocultured wells were collected after removing cell debris via centrifugation. The supernatants were diluted 3–5 times before evaluating cytokines using ELISA kits according to the manufacturer’s instructions. The absorbance was detected on Cytation 3 (BioTek). Standard curves were plotted as per standards provided by the relevant kits, and the cytokine concentrations were calculated by multiplying the absorbance with the dilution ratios.

### Flow cytometry (FACS)

For surface staining, 5 × 10^5^ cells were collected and washed twice with PBS. Single-cell suspensions were then stained using the LIVE/DEAD™ Fixable Dead Cell Stain Kit (Life Technologies, USA) and antibodies against indicated surface markers, directly.

For intracellular staining, 1 × 10^6^ cells were collected and washed twice with PBS. Single-cell samples were treated with the LIVE/DEAD™ Fixable Dead Cell Stain Kit and antibodies against surface markers for 30 min at 4℃. Then, the cells were permeabilized using the Intracellular Staining Perm Wash Buffer (421002, BioLegend) and stained with antibodies against intracellular proteins. Isotype control antibodies were used for the gating strategy.

For CAR T cell proliferation analysis in vitro, 1 × 10^6^ CAR T cells were co-cultured with OSRC-2 cancer cells for 48–72 h. The cells were then collected using a pipette, washed with PBS, and stained with antibodies against human CD3 and CD45. Subsequently, the cells were permeabilized using a nuclear membrane disruption reagent, followed by staining with an anti-Ki67 antibody. Samples were detected using the FACS Canto II cell analyzer (Becton-Dickinson), and data were analyzed with the FlowJo V10 software.

### Tumor models and treatment

Male NOD-Prkdc^scid^Il2rg^null^ (NPG) mice (aged, 6–8 weeks) purchased from Vitalstar Biotechnology were maintained in the specific pathogen-free animal facilities of the Experimental Animal Center of Xuzhou Medical University (Xuzhou, P.R. China).

For a renal orthotopic xenograft tumor model, 5 × 10^5^ OSRC-2^Luc+^ cells were injected into the subrenal capsule of NPG mice. These mice were divided into different groups based on the average tumor burden after 10 days as indicated. The mice were injected with 3 × 10^6^ of the respective T cells via a tail vein. Tumor progression was monitored via luminescence imaging with an IVIS system every week, and CAR T cells in the blood were analyzed on weeks 1 using flow cytometry. The overall survival of NPG mice was recorded, and the experiment was terminated when all mice died. To assess the abundance of CAR T cells in tumors, mice were sacrificed one week after CAR T cell infusion. Tumor tissues were then harvested, and the number of infiltrated CAR T cells was analyzed by flow cytometry. Absolute Counting beads (Invitrogen, C36950) were employed to determine the absolute T cell count.

For a subcutaneous tumor model, 2 × 10^6^ OSRC-2 cells were injected into the right dorsal flank region of NPG mice. These mice were randomly assigned to two groups on day 7 or 14: CAIX-HVEM WT or CAIX-HVEM MUT CAR T cells. The mice were injected with 1 × 10^7^ of the respective CAR T cells via a tail vein. For the groups received CAR T cell on day 14, the extracted tumors were dissected into pieces and added to a digestion solution containing 1 mg/mL collagenase I (VIC079, VICMED) and 0.1 mg/mL DNase I (10104159001, Roche). The digested tumor samples were transferred to gentleMACS C tubes and processed into single-cell suspensions using Miltenyi gentleMACS dissociator at 37℃. Cytotoxicity and cytokine release were analyzed using RTCA and ELISA, respectively, after the tumor-infiltrating T cells were sorted using flow cytometry.

### Metabolic assays

The oxygen consumption rate (OCR) and extracellular acidification rate (ECAR) were assessed using the Seahorse XFp analyzer (Agilent). CAR T cells were isolated by flow cytometry, either from the in vitro co-culture with cancer cells or from tumor-infiltrating populations in a subcutaneous tumor model, and then resuspended in Seahorse XF RPMI medium (extra 1 mM sodium pyruvate, 1 mM L-glutamine, and 25 mM glucose for OCR; no glucose was added for ECAR), seeded into XF96 cell culture microplates (Agilent) at the concentration of 2 × 10^5^ cells/well, centrifugated with 400 g, 5 min, and incubated for over 60 min at 37℃ without CO_2_ to ensure their sedimentation at the bottom of the plates, followed by basal OCR and ECAR assessment for 20 min. In addition, the cells were treated with 1.5 µM oligomycin (HY-N6782, MCE), 1.0 µM carbonyl cyanide p-trifluoromethoxy phenylhydrazone (FCCP; HY-100410, MCE), 40 nM rotenone (HY-B1756, MCE), and 1 mM antimycin A (A8674, Sigma) every 20 min to detect the dynamic OCR. Meanwhile, cells were also treated with 10 mM glucose (1181302, Sigma), 1.5 µM oligomycin, and 50 mM 2-deoxy-D-glucose (HY-13966, MCE) every 20 min to measure the glycolytic capacity.

### Protein extraction and Western blotting

CAR T cells were cocultured with OSRC-2 cells at an E: T ratio of 5:1 in 10-cm plates with X-VIVO 15 medium containing rhIL-2 for the indicated times. The CAR T cells were collected and lysed in lysis buffer (50 mM Tris-HCl, pH 7.5, 150 mM NaCl, 1% Triton X-100, 0.5% deoxycholate, 0.1% SDS, 10% glycerol, 1 mM EDTA, and 1 mM EGTA) containing a protease inhibitor mixture for 30 min at 4℃. The protein samples were purified via centrifugation and stored at − 80℃.

The protein samples were quantified using a BCA kit (KGB2101, KeyGEN BioTECH), separated via SDS-PAGE, and transferred to nitrocellulose membranes. The membranes were blocked with 5% nonfat milk, incubated with primary antibodies overnight at 4℃, and then treated with secondary antibodies at room temperature for 1 h, followed by visualization on a chemiluminescence device (Tanon 4600).

### Co-immunoprecipitation (Co-IP)

CAR T cells were cocultured with OSRC-2 cancer cells at an E: T ratio of 5:1 for the indicated times. The CAR T cells were collected and lysed in lysis buffer (50 mM Tris-HCl, pH 7.5, 150 mM NaCl, 0.5% Triton X-100, 10% glycerol, 1 mM EDTA, and 1 mM EGTA) containing a protease inhibitor mixture for 30 min at 4℃ [14]. The cell lysates were purified via centrifugation. The supernatants were incubated with anti-c-Myc magnetic beads (HY-K0206, MCE) at 4℃ overnight, and the beads were washed and collected using a magnetic stand (HY-K0200, MCE). The proteins were eluted by boiling the supernatant for 5 min in 50–80 µL SDS loading buffer. The samples were subjected to shotgun mass spectrometry (MS) or Western blotting.

### RNA isolation and real-time quantitative PCR (qRT-PCR)

Total RNA from CAR T cells was extracted using the TRIzol reagent, and the concentration was measured using NanoDrop 2000. RNA (500 ng) was used for reverse transcription using the PrimeScript RT Master Mix (RR036, Takara). Meanwhile, gene expression was detected via qRT-PCR using the SYBR Green reagent (Q111-02, Vazyme) on LightCycler 96 (Roche), with β-actin as the control for normalization. The relative gene expression was analyzed using 2^−△△CT^. The information of primer sequences is provided in supplementary Table S4.

### RNA sequencing and gene set enrichment analysis (GSEA)

Total RNA from CAR T cells was extracted using the TRIzol reagent following the manufacturer’s instructions. Next, RNA was sequenced as described previously [13]. In brief, mRNA was purified from total RNA (5 µg) using Dynabeads Oligo (dT) (Thermo Fisher, CA, USA) and fragmented into short fragments using divalent cations with the Magnesium RNA Fragmentation Module (NEB, MA, USA). The cleaved RNA fragments were reverse-transcribed to form cDNA with SuperScript™ II Reverse Transcriptase (Invitrogen, CA, USA). The cDNA was used to synthesize U-labeled double-stranded DNA with *Escherichia coli* DNA polymerase I (NEB, MA, USA), RNase H (NEB, MA, USA), and dUTP solution (Thermo Fisher, CA, USA). Finally, 2 × 150 bp paired-end sequencing (PE150) was performed on the Illumina Novaseq™ 6000 (LC-Bio Technology CO., Ltd., Hangzhou, China) following the vendor’s recommended protocol.

GSEA was performed using the GSEA (v4.1.0) and MSigDB software to identify a set of genes in specific KEGG pathways that were significantly differently expressed between CAIX-HVEM-CAR T and CAIX-4-1BB-CAR T cell treatment. The enrichment scores and *p* value were calculated with default parameters. KEGG pathways with NES > 1 or NES < − 1 and NOM *p*-value < 0.05 were considered significant in the two groups.

### Shotgun liquid chromatography–tandem MS (LC–MS)

Sediments from co-IP samples were released by boiling for 5 min with 100 mM DTT. Next, 200 µL UA buffer (8 M Urea, 150 mM Tris-HCl, pH 8.5) was added to the samples and filtered in a 30-KD ultrafiltration tube via centrifugation at 14,000 × g for 15 min. Then, 100 µL of 25 mM NH_4_HCO_3_ was added and centrifuged at 14,000 × g for 15 min twice. Finally, 40 µL trypsin buffer (4 µg of trypsin in 40 µL of 25 mM NH_4_HCO_3_ buffer) was added, followed by vortexing for 1 min and incubation for 16–18 h at 37℃. The solution was centrifuged at 14,000 × g for 15 min, and the peptides in the supernatant were quantified using NanoDrop 2000.

In LC, the samples were separated using the Easy nLC system with buffer A (0.1% formic acid aqueous solution) and buffer B (0.1% formic acid + 80% acetonitrile aqueous solution). The Acclaim PepMap RSLC (50 μm × 15 cm, nano viper, P/N164943) column was used for LC. The samples were loaded with the increasing concentration of buffer B (i.e., 0%–6% from 0 to 5 min, 6%–28% from 5 to 45 min, 28%–38% from 45 to 50 min, 38%–100% from 50 to 55 min, and 100% from 55 to 60 min). The samples were analyzed on a Q Exactive instrument after chromatographic separation for 60 min. The detection method was positive ions, scanning range of the parent ion was 350–1800 m/z, primary MS resolution was 70,000, and secondary MS resolution was 17,500. The data were analyzed using the Proteome Discoverer 2.1 software (Thermo Scientific).

### Statistical analysis

Statistical analysis was performed using Graphpad Prism 8.1 software, and data are presented as the mean ± SD, as indicated in the figure legends. Statistical difference between mean values was determined using either unpaired Student’s t-test or one-way analysis of variance (ANOVA). Survival curves were analyzed using the log-rank test. In all cases, a significant difference was defined as *P* < 0.05.

### Data availability statement

The data generated in this study are available within the article and the supplementary data files.

## Results

### HVEM-based CAR T cells exhibit superior efficacy against solid tumors compared to 4-1BB-based CAR T Cells

**Fig. 1:**
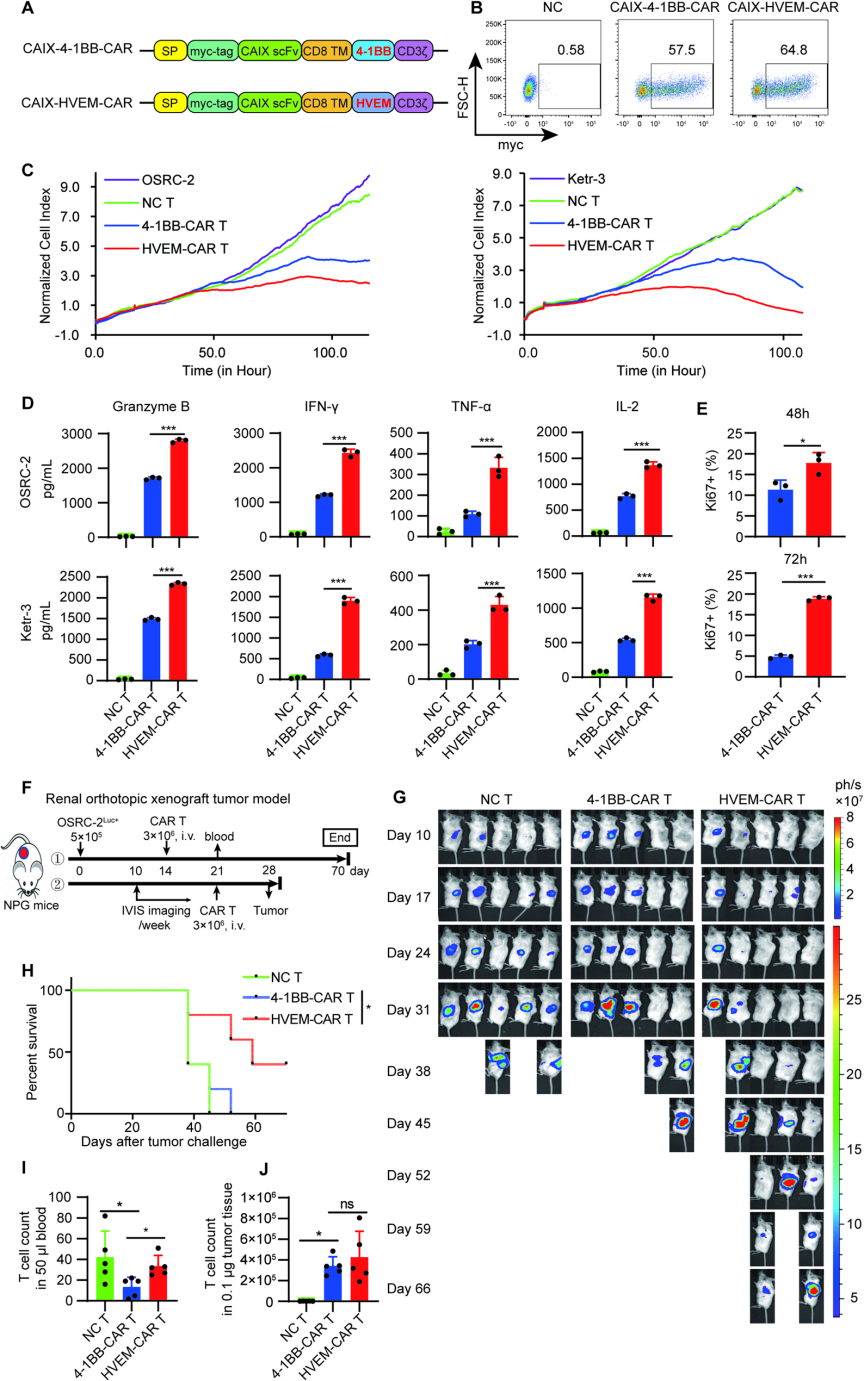
HVEM-based CAR T Cells Exhibit Superior Efficacy Against Solid Tumors Compared to 4-1BB-based CAR T Cells. **A** Schematic diagram illustrating the construction of CAIX-CARs for this study. An anti-human CAIX scFv was used to engineer second-generation CAR constructs with the 4-1BB or HVEM CSD. **B** CAIX-CAR expression in CAR T cells were determined by flow cytometry. **C** Cytotoxicities of CAIX-CAR T cells with the 4-1BB or HVEM CSD against CAIX-positive human renal cancer cells, OSRC-2 (Left) and Ketr-3 (Right), were analyzed by real-time cellular analysis (RTCA). Target cells were seeded at 3,000 cells/well (for OSRC-2 cells) or 5,000 cells/well (for Ketr-3 cells) in an E-plate and CAR T cells were added at an E: T of 1:5 when tumor cells reached the logarithmic growth phase. Graphs show the relative cancer cell number and NC T indicates non-transfected control T cells. **D** Cytokines (granzyme B, IFN-γ, TNF-α, and IL-2) secreted by CAR T cells in response to OSRC-2 (Upper) or Ketr-3 (Bottom) stimulation. NC T cells or CAR T cells with the 4-1BB or HVEM CSD were cocultured with target cells at an E: T of 1:5 for 24 h; cytokines in the culture media were quantified using ELISA. **E** To assess CAR T cell proliferation in response to target cancer cell stimulation, 4-1BB- or HVEM-CAIX-CAR T cells were co-cultured with OSRC-2 cancer cells for 48 h (Upper) or 72 h (Bottom), the Ki67 expression was then detected using flow cytometry. **F**–**J** NPG mice were injected with 5 × 105 OSRC-2Luc+ cells in the subrenal capsule to establish the renal orthotopic xenograft model. The mice were divided into three groups based on the average tumor burden and received either 3 × 106 NC T, CAIX-4-1BB-, or CAIX-HVEM-CAR T cells (*n* = 5) on day 14 for analysis of antitumor efficacy, or on day 21 for analysis of tumor-infiltrating T cell counts, respectively. Experiments were terminated at day 70 (**F**). The therapeutic efficacy of CAIX-CAR T cells was evaluated via luminescence imaging using the IVIS system. Images of luciferase intensity from different groups are presented (**G**). Overall survival curves of tumor-bearing mice after CAR T therapy were recorded (**H**). The cell count of human T cells in the peripheral blood of NPG mice on week 1 were detected using flow cytometry (**I**). **J** Tumor-infiltrating human T cell counts were determined using flow cytometry one week after CAR T cell infusion. Data (**E**) are presented as mean ± SD, and analyzed via t-test. Data (**D**, **I**, **J**) are presented as mean ± SD, and analyzed via one-way ANOVA. Differences between survival curves (**G**) were analyzed using the log-rank test. *, *P* < 0.05; **, *P* < 0.01; ***, *P* < 0.001; ns, not significant for indicated comparison

Our previous studies demonstrated that HVEM-CAR T cells exhibited superior anti-solid tumor efficacy compared to 4-1BB-CAR T cells [9]. To further confirm this observation and explore the mechanism underlying HVEM-driven CAR T cells, we generated second-generation CAR T cells with either 4-1BB or HVEM as the co-stimulatory domain (CSD) targeting the renal cancer antigen CAIX (Fig. 1A). CAR T cells were prepared using lentivirus transfection, ensuring comparable CAR expression across all constructs (Fig. 1B). The positive ratios of CAR were standardized for consistency in all experiments conducted in this study. OSRC-2 and Ketr-3, both CAIX-positive renal cancer cell lines [15], were used as target cancer cells. Real-time cell analysis (RTCA) demonstrated that HVEM-CAR T cells exhibited stronger cytotoxicity against cancer cells than 4-1BB-CAR T cells (Fig. 1C). ELISA results indicated that HVEM-CAR T cells released higher levels of cytokines, including granzyme B, IFN-γ, TNF-α, and IL-2, in response to both cell lines (Fig. 1D), Additionally, the proliferative capacity of CAR T cells upon tumor cell stimulation was analyzed by flow cytometry measuring Ki67 expression, which further revealed that HVEM-CAR T cells proliferated significantly more than 4-1BB-CAR T cells (Fig. 1E), suggesting that HVEM CSD-based CAR T cells have superior effects against cancer cells in vitro. CAR T cell exhaustion and memory phenotypes also been analyzed using flow cytometry (FACS) with or without tumor stimulation, while no significant differences were observed between HVEM-CAR T cells and 4-1BB-CAR T cells (Supplementary Fig. S1).

Orthotopic tumor models have been extensively utilized to replicate the biological characteristics of solid tumor growth observed in patients [16]. To evaluate the therapeutic efficacy of HVEM-CAR T cells against orthotopic tumors, OSRC-2^Luc+^ cells were injected beneath the kidney capsule of NPG mice, followed by CAR T cell treatment as indicated (Fig. 1F). In vivo imaging and overall survival curves showed that HVEM-CAR T cells exhibited enhanced antitumor activity compared to 4-1BB-CAR T cells (Fig. 1G, H). Furthermore, HVEM-CAR T cells demonstrated increased proliferation in the blood of tumor-bearing mice (Fig. 1I). Unexpectedly, a relatively high level of NC T cells was detected in the peripheral blood in this model. We hypothesized that the lack of a CAR in these NC T cells prevented their efficient trafficking to tumor sites, resulting in their accumulation in the circulation. To directly compare the abundance of NC T cells and CAR T cells with different costimulatory domains in tumor tissue, we quantified tumor-infiltrating lymphocytes by flow cytometry one week after treatment in an orthotopic renal cancer model. The results revealed that HVEM-CAR T cells constituted the most abundant T cell population within the tumors. In contrast, the number of NC T cells was substantially lower than that of either CAR T group (Fig. 1J). This suggests that the elevated presence of NC T cells in the periphery is attributable to their lack of tumor-targeting specificity, which causes them to remain in circulation rather than infiltrating the tumor tissue. Collectively, these findings indicate that HVEM-CAR T cells possess superior therapeutic efficacy compared to 4-1BB-CAR T cells in both in vitro and in vivo settings.

### HVEM CSD activates stronger TNF signaling pathways and metabolic activities in CAR T cells

**Fig. 2:**
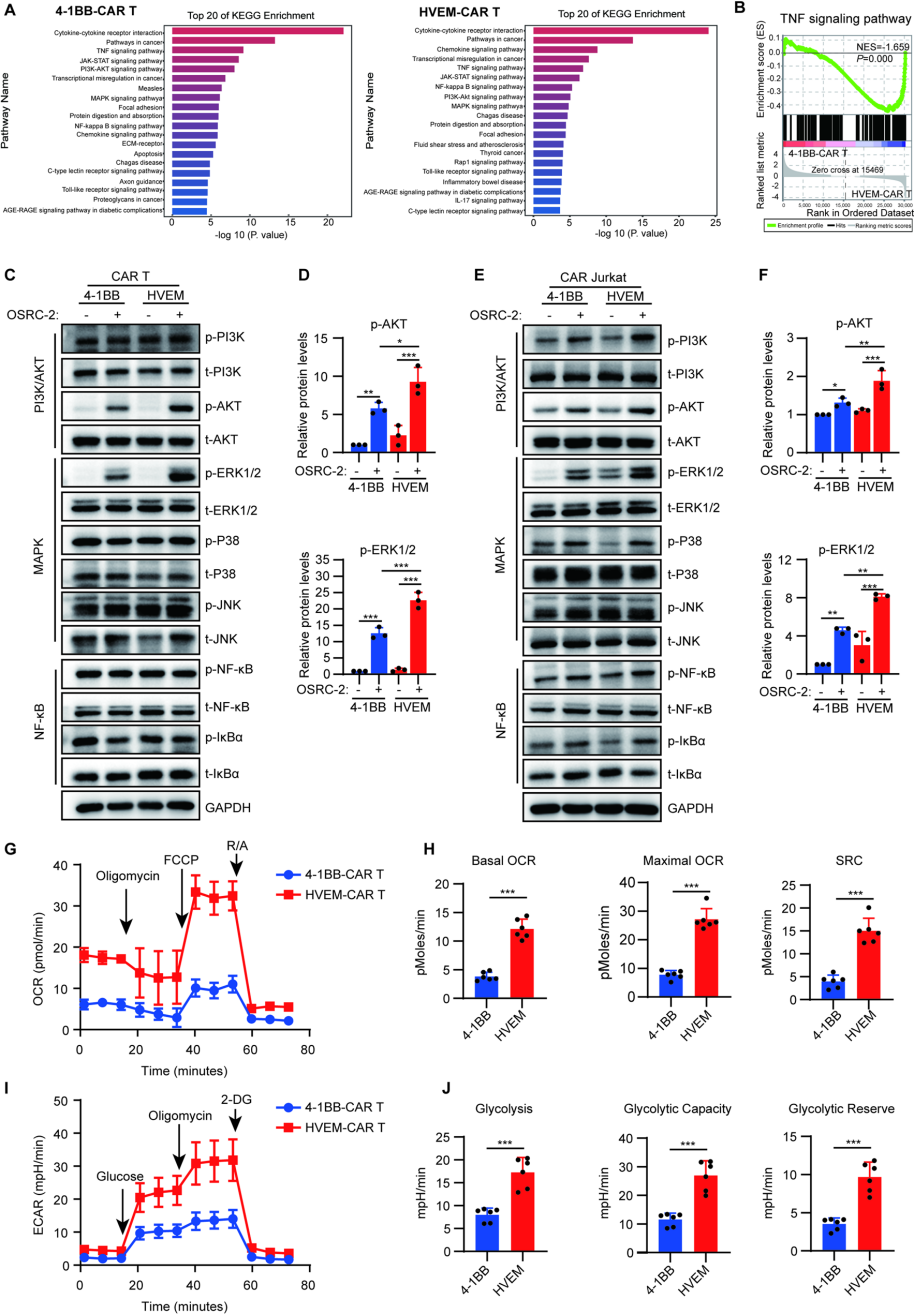
HVEM CSD activates a stronger TNF signaling pathway to enhance metabolic activities in CAR T cells. (**A**) KEGG enrichment performed to analyze CAIX-4-1BB (Left) or CAIX-HVEM (Right) CAR T cell-activated signaling pathways with CAIX antigen stimulation. Top 20 pathways are shown. CAIX-CAR T cells with the 4-1BB or HVEM CSD were stimulated with rhCAIX protein for 24 h and then subjected to RNA sequencing. (**B**) GSEA data revealing the enrichment of TNF signaling pathway in the transcriptome of 4-1BB-CAR T and HVEM-CAR T cells stimulated with CAIX antigen. (**C**) TNF signaling downstream cascades in CAR T cells detected using Western blotting. CAIX-4-1BB or CAIX-HVEM-CAR T cells cocultured without (−) or with (+) OSRC-2 cells for 5 min were collected for detecting molecules involved in the TNF signaling pathways, including PI3K/AKT, MAPK, and NF-κB signaling pathway. (**D**) Quantifications of the AKT and ERK1/2 of three independent experiments in (**C**), Quantifications of other signaling molecules were provided in Supplementary Fig. 1. (**E**) TNF signaling downstream cascades in CAR Jurkat cells detected using Western blotting. CAIX-4-1BB or CAIX-HVEM-CAR Jurkat cells cocultured without (−) or with (+) OSRC-2 cells for 5 min were collected for detecting molecules involved in TNF signaling pathways. (**F**) Quantifications of the AKT and ERK1/2 of three independent experiments in (**E**), Quantifications of other signaling molecules were provided in Supplementary Fig. 1. (**G**-**J**) The OCR and ECAR of CAIX-4-1BB- or CAIX-HVEM-CAR T cells in response to target cancer cell stimulation were determined by Seahorse assay. The CAR T cells were co-cultured with OSRC-2 cells for 24 hours. OCR of the CAR T cells was analyzed following the sequential addition of Oligomycin, FCCP, and rotenone/antimycin A (R/A) as indicated (**G**), the levels of Basal OCR, Maximal OCR, SRC were quantified (**H**). ECAR of the CAR T cells was analyzed following the sequential addition of Glucose, Oligomycin, and 2-DG as indicated (**I**), the levels of Glycolysis, Glycolytic Capacity, and Glycolytic Reserve were quantified (**J**). Data (**D**, **F**) are presented as mean ± SD, and analyzed via one-way ANOVA. Data (**H**, **J**) are presented as mean ± SD, and analyzed via t-test. *, *P* < 0.05; **, *P* < 0.01; ***, *P* < 0.001 for indicated comparison

To investigate the underlying molecular mechanisms responsible for the superior antitumor efficacy of HVEM-CAR T cells, both HVEM-CAR T and 4-1BB-CAR T cells were stimulated with recombinant antigen CAIX and subjected to RNA sequencing. The kyoto encyclopedia of genes and genomes (KEGG) enrichment analysis revealed that similar downstream signaling cascades were activated in both HVEM-CAR T and 4-1BB-CAR T cells, including TNF, Janus kinase/signal transducer and activator of transcription (JAK/STAT), phosphatidylinositol 3-kinase (PI3K)/protein kinase B (PKB/AKT), MAPK, and NF-κB pathways, etc. (Fig. 2A). Given that the downstream components of the TNF signaling pathway encompass multiple signaling networks, including PI3K/AKT, MAPK, and NF-κB pathways [17]. Therefore, we further examined the differential activation of the TNF signaling pathway between these two CAR T cell types using Gene Set Enrichment Analysis (GSEA). The results indicated a stronger activation of the TNF signaling pathway in HVEM-CAR T cells (Fig. 2B).

The involvement of key signaling molecules has also been assessed through Western blot analysis. The results demonstrated that, upon target cell stimulation, the phosphorylation levels of AKT and ERK1/2 were significantly higher in HVEM-CAR T cells than in 4-1BB-CAR T cells (Fig. 2C, D, and Supplementary Fig. S2A). The Jurkat cell line, a widely used model for investigating intracellular signaling during T cell activation [18], was employed to further validate these findings. 4-1BB-CAR T and HVEM-CAR T cells targeting CAIX were generated in Jurkat cells, referred to as 4-1BB-CAR Jurkat and HVEM-CAR Jurkat, respectively (Supplementary Fig. S3). Western blot analysis of signaling molecules involved in the PI3K/AKT, MAPK, and NF-κB pathways showed that, upon target cell stimulation, the phosphorylation levels of AKT and ERK1/2 were significantly elevated in HVEM-CAR Jurkat cells compared to 4-1BB-CAR Jurkat cells (Fig. 2E, F and Supplementary Fig. S2B). Together, these data suggest that in CAR T cells, HVEM CSD activation leads to a more robust TNF signaling pathway.

Metabolic fitness is crucial for CAR T cells to exert their effector functions. To meet the energy demands of cell proliferation and cytokine secretion, activated effector T cells increase both oxidative phosphorylation and aerobic glycolysis [19, 20]. both AKT and ERK1/2 are known to participate in the regulation of cellular metabolism [21, 22]. Concurrently, our previously published findings, along with reports from other groups, have indicated that HVEM-CAR T cells exhibit superior metabolic activity with tumor antigen stimulation compared to 4-1BB-CAR T cells [9, 23]. To further delineate the metabolic differences between HVEM-CAR T and 4-1BB-CAR T cells in this study, we assessed their metabolic profiles using Seahorse assay after co-culture with target tumor cells in vitro. The results demonstrated that HVEM-CAR T cells simultaneously possessed higher levels of both OXPHOS parameters, including basal OCR, maximal OCR, and spare respiratory capacity (SRC) (Fig. 2G, H), and glycolytic parameters, such as Glycolysis, Glycolytic Capacity, and Glycolytic Reserve (Fig. 2I, J), consistent with our prior in vivo observation [9], further confirming that upon tumor antigen stimulation, HVEM-CAR T cells exhibit superior metabolic activity both in vitro and in vivo, which translates into enhanced antitumor efficacy.

### Enhanced TRAF binding activity of HVEM CSD drives stronger TNF signaling

TNF receptor-associated factors (TRAFs) act as an intermediary in the TNF signaling pathway [24]. They help transmit signals from the TNF receptors to other molecules inside the cell, which can lead to various cellular responses such as inflammation and immune response regulation [17]. The costimulatory molecules 4-1BB and HVEM both belong to the tumor necrosis factor receptor superfamily (TNFRSF) [25]. TNFRSF costimulatory molecules play a crucial role in the immune response. When activated, TNFRSF costimulatory molecules recruits TRAFs to the cytoplasmic domain of the receptor. TRAFs act as signal transducers by forming protein complexes and initiating a cascade of downstream signaling events [17, 26].

**Fig. 3:**
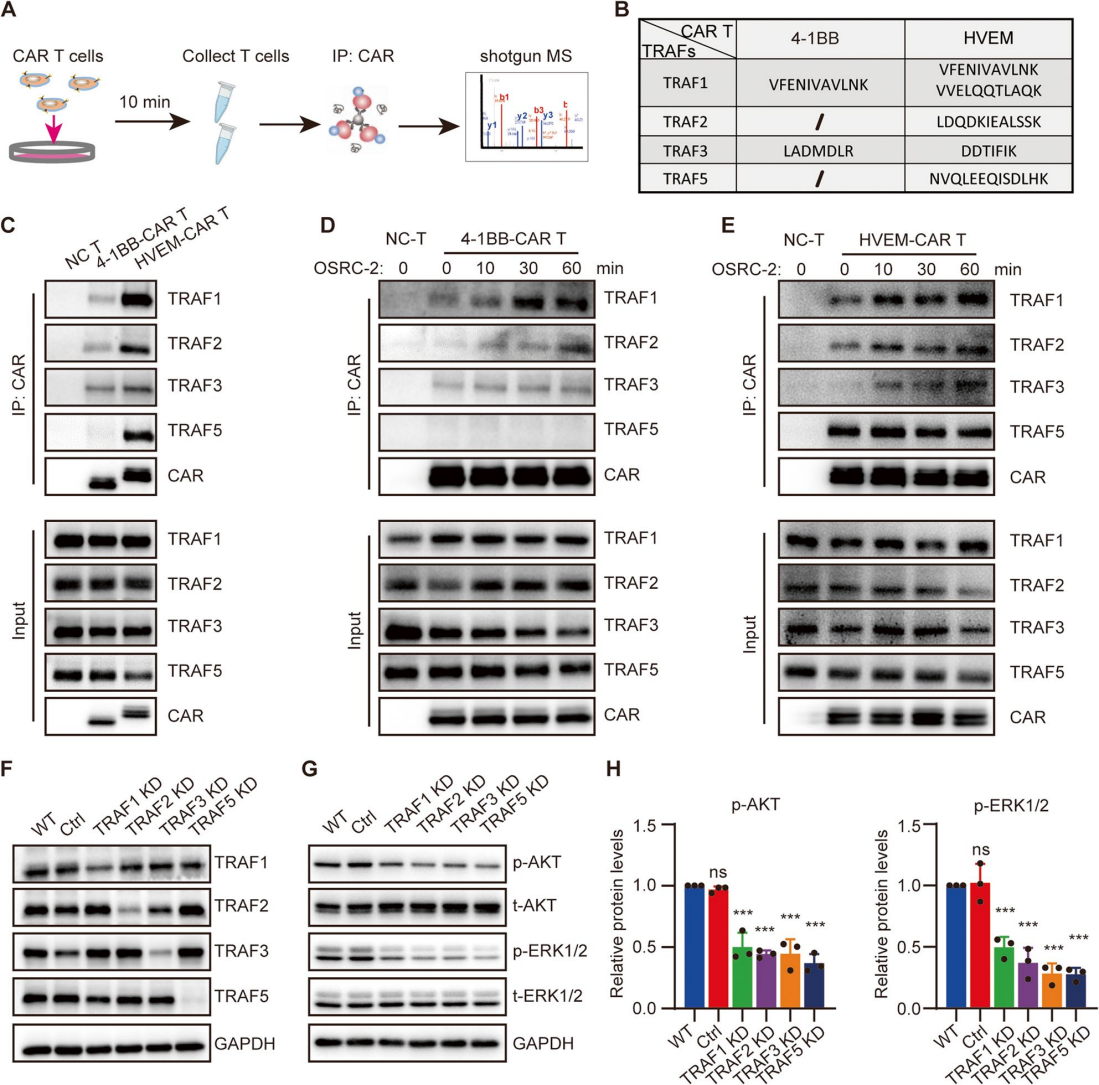
Enhanced TRAF Binding Activity of HVEM CSD Drives Stronger TNF Signaling. **A** Schematic diagram of the experimental design of combined Co-IP and shotgun MS. CAIX-4-1BB or CAIX-HVEM-CAR T cells were cocultured with OSRC-2 cancer cells for 10 min. Next, the CAR T cells were collected and lysed, CAR and associated proteins were enriched via Co-IP, and the sediments were analyzed using shotgun MS. **B** TRAF peptides identified in the sediment of 4-1BB or HVEM-CAR via shotgun MS. **C** Association between TRAFs and 4-1BB-CAR or HVEM-CAR determined using co-IP. CAIX-4-1BB- or CAIX-HVEM-CAR T cells were cocultured with OSRC-2 cancer cells for 10 min. Then, CAR T cells were collected and lysed for co-IP assay using anti-c-Myc magnetic beads. TRAF1, TRAF2, TRAF3, and TRAF5 were detected in sediments via Western blotting. (**D**, **E**) Time-dependent association between TRAFs and 4-1BB-CAR **D** or HVEM-CAR (**E**) determined using Co-IP. CAIX-4-1BB- or CAIX-HVEM-CAR T cells were cocultured with OSRC-2 cancer cells for the indicated times, followed by the collection and analysis of CAR T cells via Co-IP assay using anti-c-Myc magnetic beads. TRAF1, TRAF2, TRAF3, and TRAF5 were detected in sediments via Western blotting. (**F**) TRAF1, TRAF2, TRAF3, and TRAF5 were knocked down in CAIX-HVEM-CAR Jurkat cells via lentivirus transfection, and the knockdown efficacy was determined via Western blotting. **G** CAIX-HVEM-CAR Jurkat cells with TRAF1, TRAF2, TRAF3, and TRAF5 deficiency were cocultured with OSRC-2 cancer cells for 5 min. Then, the CAR Jurkat cells were collected and lysed, and the level of phosphorylated (p)-AKT, p-ERK, total (t)-AKT, and t-ERK was assessed through Western blotting. **H** Quantifications of the AKT and ERK1/2 of three independent experiments in (**G**). Data (**H**) are presented as mean ± SD, and analyzed via one-way ANOVA. ***, *P* < 0.001; ns, not significant for indicated comparison to WT

To investigate the differences in the binding of TRAFs by HVEM-CAR and 4-1BB-CAR, after CAR T cells being stimulated by the target cells (OSRC-2) for 10 min, CAR and associated proteins in CAR T cells were enriched using Co-IP assay and the sediments were analyzed using Shotgun LC-MS (Fig. 3A). Only TRAF1 and TRAF3 were detected in 4-1BB-CAR T samples, whereas TRAF1, TRAF2, TRAF3, and TRAF5 were detected in HVEM-CAR T samples (Fig. 3B). The TRAF peptides identified in the sediments using Shotgun MS are shown in supplementary Figure S4. Previous studies have reported that the costimulatory molecule 4-1BB can recruit TRAF2 [27, 28], which is inconsistent with our Shotgun LC-MS results. A possible explanation for this discrepancy is the insufficient sensitivity of Shotgun LC-MS detection.

To further confirm the TRAFs that bind to CAR, after CAR T cells being stimulated by OSRC-2 cells for 10 min, CAR-TRAF complex was determined via CO-IP/Western blotting experiments. The results showed that TRAF1, TRAF2, and TRAF3 were coimmunoprecipitated in 4-1BB-CAR T samples, whereas TRAF1, TRAF2, TRAF3, and TRAF5 were coimmunoprecipitated in HVEM-CAR T samples. Furthermore, the abundance of TRAF1, TRAF2, and TRAF3 in HVEM-CAR T samples were significantly higher than them in 4-1BB-CAR T samples (Fig. 3C).

To further detect the specific interactions between TRAFs and 4-1BB-CAR or HVEM-CAR, 4-1BB-CAR T or HVEM-CAR T cells were stimulated with OSRC-2 cells for different times. The results showed that the binding strength of TRAF1, TRAF2, and TRAF3 to 4-1BB-CAR was enhanced in a time-dependent manner. However, no binding of TRAF5 to 4-1BB-CAR was detected at any of the time points (Fig. 3D). In HVEM-CAR T cells, the binding strength of TRAF1, TRAF2, and TRAF3 to HVEM-CAR was enhanced in a time-dependent manner. Furthermore, TRAF5 and HVEM-CAR exhibited a strong binding affinity in the absence of stimulation, and the binding strength further enhanced upon tumor antigen stimulation (Fig. 3E).

To explore the critical role of TRAFs in the TNF signal transduction in HVEM-CAR T cells, HVEM-CAR Jurkat cell lines with TRAF1, TRAF2, TRAF3, and TRAF5 knockdown were established using specific shRNAs. The expression levels of TRAF1, TRAF2, TRAF3, and TRAF5 were all significantly reduced in their respective knockdown cell lines (Fig. 3F). In addition, the phosphorylation levels of AKT and ERK1/2 in the cells were significantly reduced after stimulation by the target cells OSRC-2 (Fig. 3G, H). In conclusion, the stronger TNF signal activated by HVEM CSD is mainly due to its stronger TRAFs binding activity.

### AVEE-mediated TRAFs binding is crucial for the antitumor function of HVEM-CAR T cells

**Fig. 4:**
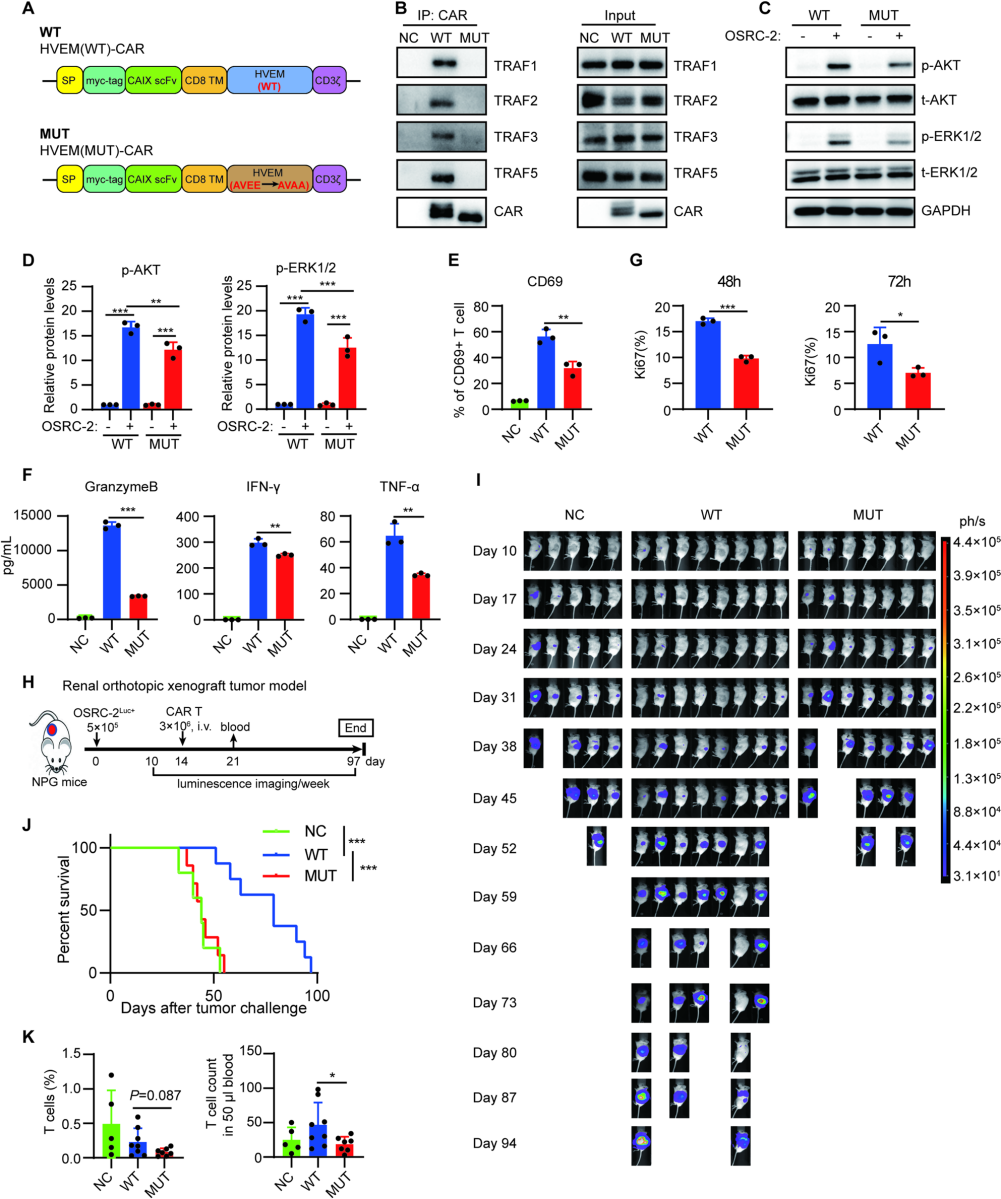
TRAF-mediated TNF signaling is crucial for the antitumor function of HVEM-CAR T cells. **A** Schematic diagram showing the structure of wild-type (WT) or AVEE mutated (MUT) HVEM-CARs. The scFv derived from a monoclonal antibody against the colon carcinoma antigen CAIX was used to engineer CAR constructs with wild type (WT) or AVEE site mutation HVEM (MUT) CSD. **B** Effect of AVEE motif on the binding of HVEM-CAR to TRAFs was determined by introducing AVEE mutation in HVEM-CAR T cells. CAR T cells were cocultured with OSRC-2 cancer cells for 10 min. The cells were collected and lysed for Co-IP assay using anti-c-Myc magnetic beads. TRAF1, TRAF2, TRAF3, and TRAF5 were detected in sediments through Western blotting. **C** Wild-type HVEM-CAR T cells (WT) or AVEE-mutated HVEM-CAR T cells (MUT) were cocultured with OSRC-2 cancer cells for 0 (−) or 5 min (+). The cells were collected and lysed for AKT and ERK1/2 activation via Western Blotting. **D** Quantifications of the AKT and ERK1/2 of three independent experiments in (C). **E** Expression of CD69 in CAIX-CAR T cells with WT or MUT HVEM CSD in response to cancer antigen stimulation. WT or MUT HVEM-CAR T cells were cocultured with rhCAIX antigen for 8 h, and CD69 expression was detected via flow cytometry. **F** Granzyme B, INF-γ, and TNF-α secreted by WT or MUT HVEM-CAR T cells in response to target cancer cell stimulation. WT or MUT HVEM-CAR T cells were cocultured with OSRC-2 cancer cells for 24 h, and granzyme B, TNF-α, and INF-γ in the coculture suspension were quantified using ELISA. **G** The proliferative capacities of WT and MUT HVEM-CAR T cells in response to target cancer stimulation. WT or MUT HVEM- CAR T cells were cocultured with OSRC-2 cancer cells for 48–72 h, the Ki67 expression was then detected using flow cytometry. **H**–**K** NPG mice were injected with 5 × 105 OSRC-2Luc+ cells in the subrenal capsule to establish a renal orthotopic xenograft model. The mice were divided into three groups based on the average tumor burden and received either 3 × 106 NC T (*n* = 5), WT (*n* = 8), or MUT HVEM-CAR T cells (*n* = 7) on day 14, respectively. Experiments were terminated when all mice died at day 97 (**H**). The therapeutic efficacy of WT or MUT HVEM-CAR T cells was evaluated via luminescence imaging using the IVIS system. Images of luciferase intensity from different groups are presented (**I**). Overall survival curves of tumor-bearing mice after CAR T therapy were recorded (**J**). Frequencies and cell count of human T cells in the peripheral blood of NPG mice on week 1 were detected using flow cytometry (**K**). Data (**D**, **E**, **G**, **K**) are presented as mean ± SD and were analyzed using one-way ANOVA. Data (**F**) are presented as mean ± SD, and analyzed via t-test. Differences between survival curves (**I**) were analyzed using the log-rank test. *, *P* < 0.05; **, *P* < 0.01; ***, *P* < 0.001 for indicated comparison

Previous reports have suggested that the conserved motif (P/S/A/T)X(Q/E)E is the binding site for TRAFs in TNFRSF [25, 29]. Based on the characteristics of the conserved motifs, the amino acid motif AVEE, which is located at positions 268–271 amino acids of HVEM (UniProt, Q92956), is predicted to be the TRAF binding site of the HVEM CSD. To confirm whether the AVEE motif is the TRAF binding site of HVEM CSD, the AVEE motif of HVEM-CAR was mutated to AVAA (Fig. 4A). The positivity rates of wild-type HVEM-CAR (WT-CAR) and mutant HVEM-CAR (MUT-CAR) expression in T cells are comparable (supplementary Fig. S5). The binding activity of MUT-CAR to TRAFs was determined by Co-IP/Western blotting experiments, and the results showed that The AVEE mutation leads to the inability of HVEM-CAR to bind to TRAFs, including TRAF1, TRAF2, TRAF3, and TRAF5 (Fig. 4B). Meanwhile, the AVEE mutation also caused the downregulation of the phosphorylation levels of AKT and ERK1/2 in HVEM-CAR T cells induced by target cell stimulation (Fig. 4C, D). Together, these data demonstrated that AVEE is the TRAFs-binding site of HVEM CSD.

Since the AVEE motif mutation can completely abrogate the binding between TRAFs and the HVEM CSD, we used both wild-type HVEM-CAR T cell and mutant HVEM-CAR T cell to study the role of TRAFs-mediated TNF signaling in the antitumor function of HVEM-CAR T. In vitro experimental results showed that the AVEE motif mutation significantly reduced the expression of the T cell activation marker CD69 and the release of the cytokines granzyme B, IFN-γ, and TNF-α in HVEM-CAR T cells (Fig. 4E, F). Furthermore, to assess the impact of the AVEE site mutation on the proliferation of HVEM-CAR T cells, we performed flow cytometry following tumor cell stimulation. The results showed that AVEE site mutation led to a significant decrease in Ki67 expression (Fig. 4G), indicating impaired proliferative capacity. For in vivo experiments, OSRC-2^Luc+^ cells were used to establish a renal orthotopic tumor model to explore the effect of AVEE mutation on the antitumor efficacy of HVEM-CAR T cells (Fig. 4H). The in vivo imaging of mice indicated that AVEE mutation decreased the antitumor efficacy compared with wild-type HVEM-CAR T cells (Fig. 4I). Likewise, AVEE mutation shortened the overall survival curves of tumor-bearing mice (Fig. 4J). Additionally, the number of CAR T cells in the peripheral blood one week after treatment indicates that the AVEE mutation reduced the survival or proliferation capacity of CAR T cells in the peripheral blood of tumor-bearing mice (Fig. 4K). These experimental results indicate that AVEE is the TRAF binding site of HVEM CSD and also suggest that TRAF-mediated TNF signaling is crucial for the antitumor function of HVEM-CAR T cells.

### TRAF-mediated TNF signaling is crucial for the infiltration, cytokine release, and cytotoxicity of HVEM-CAR T cells in tumor tissue

**Fig. 5:**
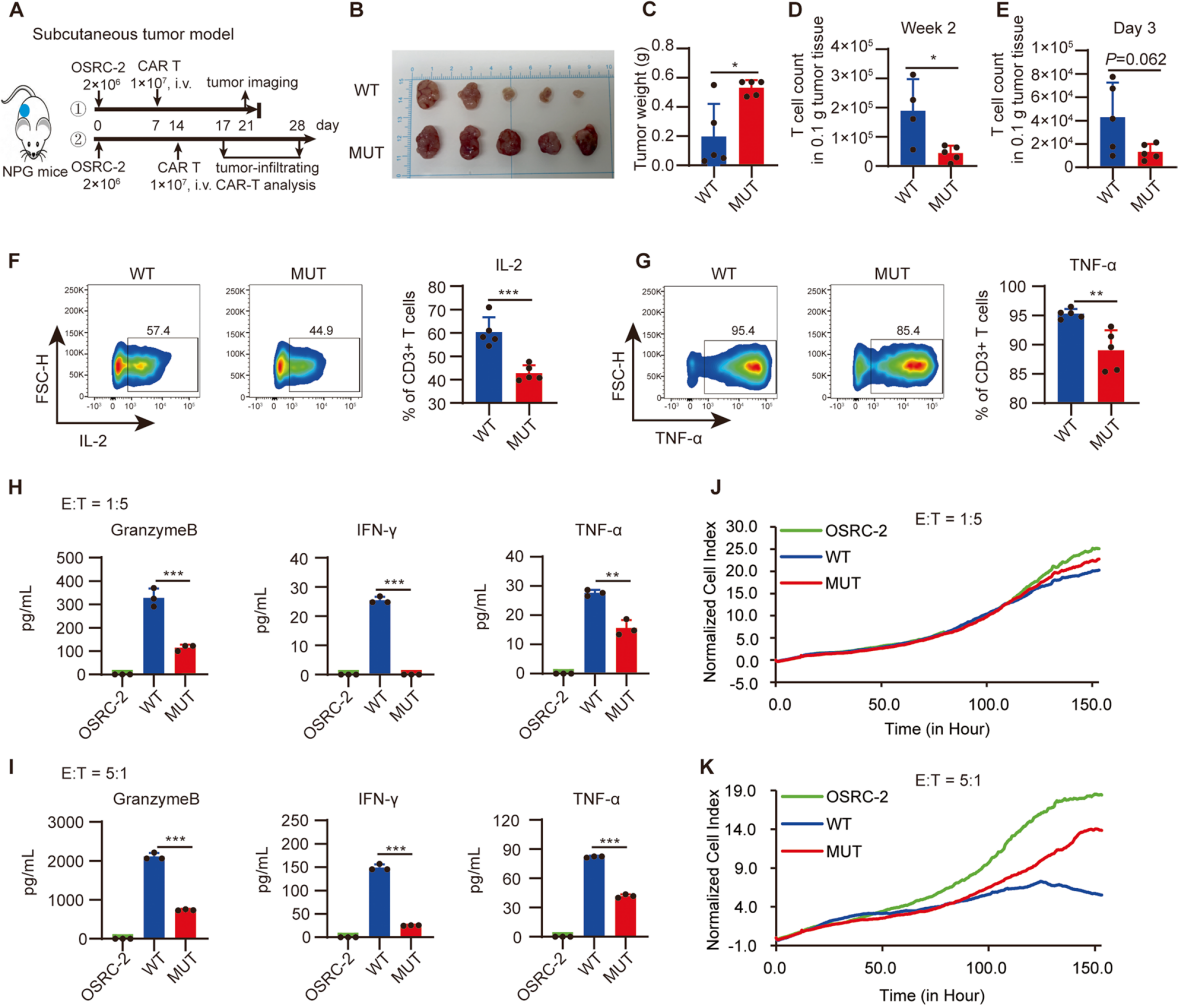
TRAF-mediated TNF signaling is crucial for the infiltration, cytokine release, and cytotoxicity of HVEM-CAR T cells in tumor tissue. **A** NPG mice were injected subcutaneously in the right dorsal flank with 2 × 106 OSRC-2 cells to establish subcutaneous tumor models. On day 7 (①) or 14 (②), the mice were randomly assigned to one of two groups (n = 5 each) that received either 1 × 107 WT or MUT HVEM-CAR T cells, respectively. The mice (①) were sacrificed two weeks after CAR T cell administration for tumor burden assessment, while, the mice (②) were sacrificed two weeks or three days after CAR T cell infusion for the analysis of tumor-infiltrating CAR T cells. Tumor images (**B**) and tumor weights (**C**) were recorded for different groups as indicated. **D**, **E** Tumor-infiltrating CAR T cell counts were determined via flow cytometry at two weeks (**D**) and three days (**E**) after CAR T cell infusion. **F**, **G** Single-cell suspension was treated with 20 ng/mL PMA, 1 µM Ionomycin, and 10 µg/mL Brefeldin A for 6 h, and IL-2 (**F**) and TNF-α (**G**) levels in tumor-infiltrating CAR T cells were determined via flow cytometry. **H**, **I** Cytokine expression by CAR T cells sorted from tumor tissues in response to target cancer cell stimulation. WT or MUT HVEM-CAR T cells sorted from tumor tissues, CAR T cells were cocultured with OSRC-2 cancer cells for 24 h in the E: T ratio of 1:5 (**H**) or 5:1 (**I**), and granzyme B, INF-γ, and TNF-α in the coculture suspension were quantified using ELISA. (**J**, **K**) Cytotoxicity of sorted CAR T cells against human renal cancer cell OSRC-2 was analyzed using RTCA. OSRC-2 cells were seeded at 3000 cells/well in an E-plate. The sorted CAR T cells from tumors were added in the E: T ratio of 1:5 (J) or 5:1 (**K**) when the target cells reached the logarithmic growth phase. Graphs show cancer cell number. Data (**C**-**G**) are presented as mean ± SD and were analyzed using the t-test. Data (**H**, **I**) are presented as mean ± SD and were analyzed using one-way ANOVA. *, *P* < 0.05; ***, *P* < 0.001 for indicated comparison

To investigate the mechanism by which TRAF-mediated TNF signaling contributes to the antitumor activity of HVEM-CAR T cells, subcutaneous tumor models were established in NPG mice using OSRC-2 cells and treated with wild-type HVEM-CAR T cells or mutant HVEM-CAR T cells, two weeks after treatment, the experiment was terminated, and tumor tissues were collected for analysis (Fig. 5A). As shown in Fig. 5B and C, the AVEE mutation significantly weakened the therapeutic effect of HVEM-CAR T cells on subcutaneous tumors. Flow cytometry analysis revealed that the AVEE mutation significantly reduced the HVEM-CAR T cell counts in tumor tissue two weeks post-infusion (Fig. 5D). The abundance of tumor-infiltrating CAR T cells is determined by their infiltration and proliferative capacities. To delineate the cause of the reduced CAR T cell numbers in tumors, we quantified them at an early time point (day 3 post-infusion). The results showed that the AVEE mutation already led to fewer CAR T cells to some extent (Fig. 5E). Combined with our prior in vitro finding that the AVEE mutation impairs proliferative response to tumor stimulation (Fig. 4G), indicating that the AVEE site plays a critical role in both the tumor infiltration and the effector expansion of HVEM-CAR T cells. Intracellular flow cytometry staining confirmed that the AVEE mutation significantly decreased the ability of HVEM-CAR T cells to release cytokines IL-2 and TNF-α in the tumor tissue (Fig. 5F, G). To further investigate the impact of intervening in TRAF-mediated TNF signaling on HVEM-CAR T cell function in tumor tissue, we sorted tumor-infiltrating CAR T cells by flow cytometry, co-cultured these CAR T cells with target cells (OSRC-2) at the 1:5 or 5:1 effector-to-target ratio for 24 h, and collected the supernatants. ELISA was used to measure the levels of Granzyme B, IFN-γ, and TNF-α released by the CAR T cells. The results showed that the AVEE mutation significantly reduced the cytokine release ability of HVEM-CAR T cells in the tumor tissue (Fig. 5H, I). Additionally, the sorted CAR T cells were also subjected to RTCA assays to assess their cytotoxicity against target cells. The intratumor CAR T cells showed minimal cytotoxicity against target cells at E: T of 1:5 (Fig. 5J), a ratio that all fresh CAR T cells were able to efficiently kill target tumor cells (Fig. 1C). These data suggest that the cytotoxicity of the CAR T cells was attenuated in solid tumor tissues. However, when the E: T ratio was increased to 5:1, the wild type HVEM-CAR T cells sorted from tumors were able to kill target cells more efficiently than AVEE mutated HVEM-CAR T cells (Fig. 5K). These findings indicate that TRAF-mediated TNF signaling is essential for the infiltration, cytokine release, and cytotoxicity of HVEM-CAR T cells in solid tumor tissues.

### Sustained metabolic activities of HVEM-CAR T cells in tumor tissue depend on the AVEE site

**Fig. 6:**
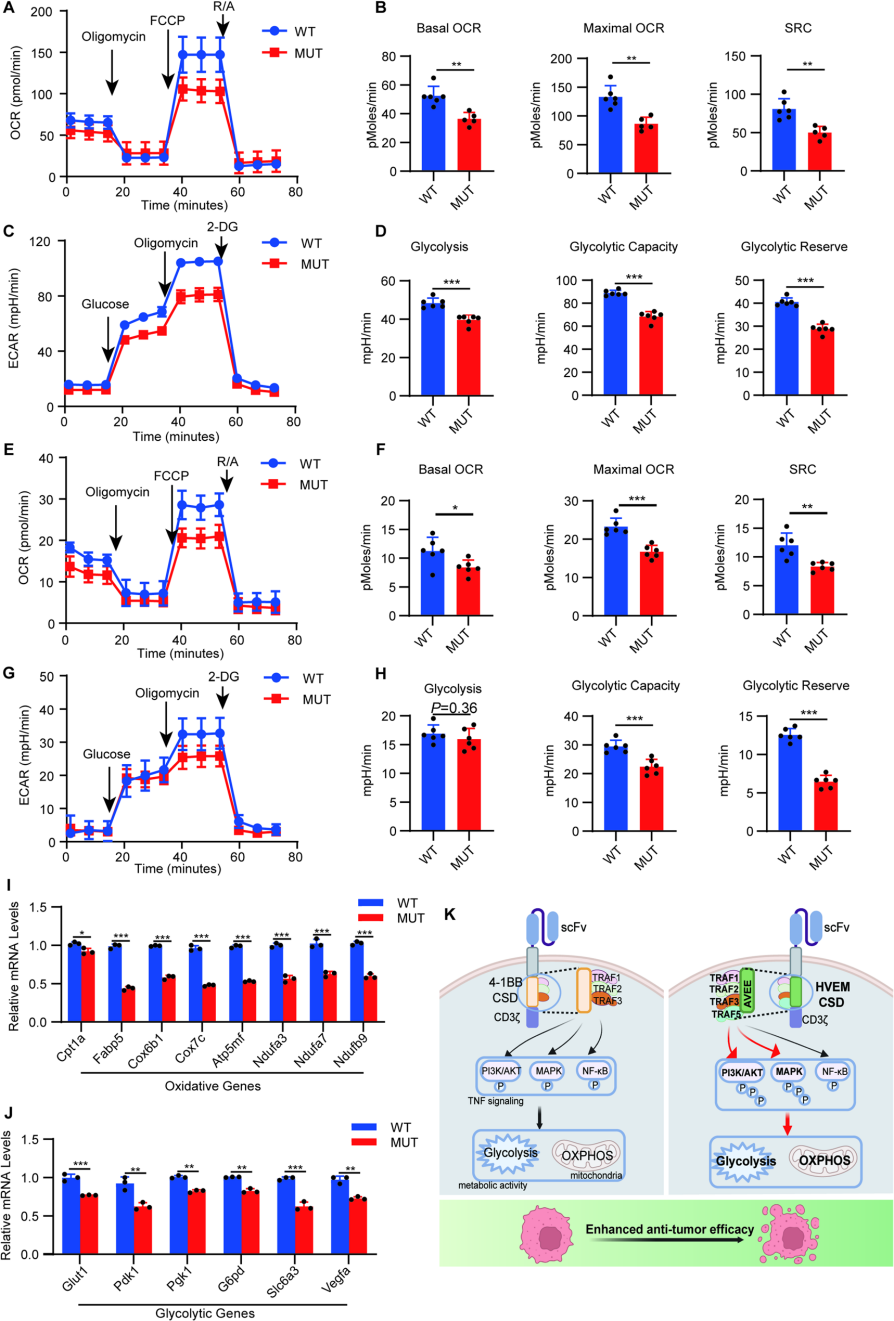
Sustained metabolic activities of HVEM-CAR T cells in tumor tissue depend on the AVEE site. **A**-**D** The OCR and ECAR of WT or MUT CAIX-HVEM-CAR T cells in response to target cancer cell stimulation were determined by Seahorse assay. The CAR T cells were co-cultured with OSRC-2 cells for 24 h. OCR of the CAR T cells was analyzed following the sequential addition of Oligomycin, FCCP, and R/A as indicated (**A**), the levels of Basal OCR, Maximal OCR, SRC were quantified (**B**). ECAR of the CAR T cells was analyzed following the sequential addition of Glucose, Oligomycin, and 2-DG as indicated (**C**), the levels of Glycolysis, Glycolytic Capacity, and Glycolytic Reserve were quantified (**D**). **E**–**H** NPG mice were injected subcutaneously in the right dorsal flank with 2 × 106 OSRC-2 cells to establish a subcutaneous tumor model. On day 14, the mice were randomly assigned to two groups (*n* = 5 each) that received either 1 × 107 WT or MUT HVEM-CAR T cells, respectively. The mice were sacrificed 2 weeks after CAR T cell administration. CAR T cells in the tumors sorted through flow cytometry were analyzed using the Seahorse assay. (**E**) OCR of tumor-infiltrating CAR T cells was determined using the Seahorse assay. **F **Summarized data showing the basal OCR, maximal OCR, and SRC of CAR T cells. **G** ECAR of tumor-infiltrating CAR T cells was determined using the Seahorse assay. **H** Summarized data showing the Glycolysis, Glycolytic Capacity, and Glycolytic Reserve of CAR T cells. **I**, **J** WT or MUT HVEM-CAR T cells were stimulated with the rhCAIX protein for 24 h. mRNA level of the oxidative genes Cpt1a, Fabp5, Cox6b1, Cox7c, Atp5mf, Ndufa3, Ndufa7, and Ndufb9 (**I**) and that of the glycolytic genes Glut1, Pdk1, Pgk1, G6pd, Slc16a3, and Vegfa (**J**) were determined using qPCR. (**K**) Model diagram of HVEM CSD promoting CAR T cell antitumor efficacy. Compared with 4-1BB-CAR T cells, HVEM CSD-driven CAR T cells recruit greater numbers of TRAF1, TRAF2, and TRAF3 molecules. Moreover, they bind to TRAF5 through the AVEE motif, thereby strongly activating the TNF signaling pathway, especially by phosphorylating AKT and ERK1/2. The enhanced phosphorylation of AKT and ERK1/2 boosts the antitumor efficacy of CAR T cells by increasing the metabolic activities of OXPHOS and glycolysis. The diagram was prepared using Biorender. Data are presented as mean ± SD and were analyzed using the t-test. *, *P* < 0.05; **, *P* < 0.01; ***, *P* < 0.001 for indicated comparison

Metabolic activity critically determines the effector functions of T cells [19, 20]. In the present study and our previous study indicated that HVEM-CAR T cells displayed both higher oxidative phosphorylation (OXPHOS) and glycolysis levels than 4-1BB-CAR T cells both in vitro and in vivo [9]. To investigate the regulatory role of the AVEE site in the metabolic activity of HVEM-CAR T cells upon antigen stimulation, we compared wild-type and mutant HVEM-CAR T cells after a 24-hour co-culture with OSRC-2 cells. Seahorse analysis of FACS-sorted T cells revealed that the MUT CAR T cells exhibited significant reductions in both oxidative phosphorylation and glycolytic activities (Fig. 6A-D). Furthermore, tumor-infiltrating CAR T cells in a subcutaneous tumor model were sorted using flow cytometry as described above, the OCR and ECAR in wild-type HVEM-CAR T cells or HVEM-CAR T cells with AVEE mutation in vivo were analyzed by Seahorse. The results revealed that basal OCR, maximal OCR, and SRC were significantly decreased in HVEM-CAR T cells with AVEE motif mutation compared with wild-type HVEM-CAR T (Fig. 6E, F). In addition, the glycolytic capacity also decreased significantly in HVEM-CAR T cells with AVEE mutation (Fig. 6G, H). To further confirm the influence of AVEE site mutation on metabolic activities of HVEM-CAR T cells, the expression of OXPHOS and glycolytic genes was determined, and the results showed that the expression of the OXPHOS genes *Cpt1a*, *Fabp5*, *Cox6b1*, *Cox7c*, *Atp5mf*, *Ndufa3*, *Ndufa7*, and *Ndufb9*, as well as that of glycolytic genes *Glut1*, *Pdk1*, *Pgk1*, *G6pd*, *Slc16a3*, and *Vegfa* [11, 30] were decreased in HVEM-CAR T cells with AVEE mutation (Fig. 6I, J). These findings indicated that sustained metabolic activities of HVEM-CAR T cells in response to cancer cell stimulation depend on the AVEE site both in vitro and in vivo.

## Discussion

The current study demonstrates that the herpesvirus entry mediator (HVEM) CSD activates a stronger TNF signaling pathway compared to the 4-1BB CSD. This enhanced activation is critical for CAR T cell function, as the strength of TNF signaling directly influences the persistence and metabolic reprogramming of CAR T cells [31, 32]. HVEM-driven CAR T cells exhibit superior antitumor efficacy against solid tumors, which can be attributed to the robust activation of TNF signaling. In contrast, CAR T cells utilizing 4-1BB CSD, though effective, show comparatively weaker signaling in response to tumor antigens. This difference in signaling strength underpins the enhanced metabolic activities observed in HVEM-CAR T cells, contributing to their superior functional performance in preclinical models.

A key finding in our study is the differential recruitment of TRAFs by the HVEM and 4-1BB CSDs. While both CSDs recruit TRAF1, TRAF2, and TRAF3, HVEM-CAR T cells additionally recruit TRAF5 via the AVEE motif, a unique feature not observed with 4-1BB-CAR T cells [25]. The binding of these TRAFs is critical for the activation of downstream TNF signaling pathways, including the PI3K/AKT, MAPK, and NF-κB pathways, which are essential for CAR T cell expansion, persistence, and functional activity [33, 34]. The increased number of TRAFs bound to HVEM-CAR T cells likely results in a more robust signaling cascade, leading to enhanced oxidative metabolism and glycolysis, which are pivotal for CAR T cell survival and function in the tumor microenvironment [35]. This study highlights the significance of TRAF recruitment in determining the strength of TNF signaling and its subsequent impact on CAR T cell efficacy.

Previous studies have established that TRAF molecules play diverse roles in determining T cell fate, including activation, differentiation, and survival [36]. However, understanding of the functions of individual TRAF members remains divergent or even contradictory. For instance, while TRAF3 is widely recognized as participating in NF-κB activation, its overexpression can inhibit CD40-mediated NF-κB signaling, indicating dual regulatory roles in both positive and negative regulation [37, 38]. In this study, we found that individual knockdown of TRAF1, TRAF2, TRAF3, or TRAF5 each led to a similar and significant reduction in AKT and ERK1/2 phosphorylation. This finding suggests a high degree of functional redundancy or interdependence among these TRAF molecules within the HVEM-mediated TNF signaling pathway. This characteristic may stem from the cooperative formation of heterotrimeric complexes by TRAF family members and their functional overlap in recruiting downstream signaling molecules, such as the IKK complex [39]. Within this framework, the HVEM signalosome operates as an integrated unit, wherein the loss of any key component (e.g., TRAF1, TRAF2, TRAF3, or TRAF5) may compromise the overall stability of the complex or impair its ability to recruit and activate downstream kinases, thereby disrupting the entire signaling cascade. This sophisticated buffering mechanism ensures signaling robustness while also implying that targeting a single TRAF may be insufficient to completely block the potent co-stimulatory signal driven by HVEM. It is particularly noteworthy that, compared to 4-1BB, HVEM recruits a broader repertoire of TRAF proteins (including TRAF5) via its unique AVEE motif and forms complexes with higher stoichiometry, which may constitute the structural basis for its enhanced signaling capacity. Further investigation into the precise stoichiometry and three-dimensional architecture of the HVEM signalosome will provide deeper insights into this cooperative redundancy mechanism and guide the development of future therapeutic strategies.

The activation of TNF signaling is crucial for the metabolic reprogramming of CAR T cells, a process that is central to their function and antitumor efficacy [40, 41]. The TNF signaling pathway regulates several metabolic pathways, including glycolysis, oxidative phosphorylation, and mitochondrial biogenesis [42, 43], which are essential for sustaining CAR T cell function in the hostile tumor microenvironment [35]. HVEM-CAR T cells, by recruiting a higher number of TRAFs and inducing stronger TNF signaling, show enhanced metabolic activity, including improved glycolysis and oxidative metabolism. This metabolic reprogramming supports the long-term persistence and anti-tumor activity of CAR T cells, particularly in solid tumors where immune evasion and metabolic suppression are common challenges [25, 44]. Our findings suggest that optimizing TNF signaling through the selection of appropriate CSDs, such as HVEM, can significantly improve the efficacy of CAR-T cell therapies by enhancing their metabolic fitness.

AVEE motif is a novel feature in the TNFRSF family and distinguishes HVEM from other CSDs such as 4-1BB [17]. The AVEE motif plays a critical role in the recruitment of TRAFs, which further strengthens the activation of TNF signaling pathways. Mutations in this AVEE motif significantly impair the association between HVEM-CAR and TRAFs, leading to a reduction in TNF signaling and a corresponding decrease in CAR T cell metabolic activity and antitumor efficacy. This discovery opens new avenues for designing CAR T cell therapies that specifically target and modulate TRAF interactions, potentially improving the efficacy of these therapies in treating solid tumors.

HVEM serves as a receptor for “LIGHT” (*l*ymphotoxin - like, with *i*nducible expression, competing with HSV *g*lycoprotein D for *H*VEM on *T* lymphocytes, TNFSF14) and lymphotoxin-α, initiating costimulatory signaling. It also acts as a ligand for immunoglobulin superfamily members BTLA (B- and T-lymphocyte attenuator, CD272) and CD160, triggering T cell coinhibitory signaling [45]. Beyond its role as a CSD in the CAR structure, recent researches have extensively explored the regulation of the HVEM-associated axis to enhance CAR T effector functions. Zhang et al. reported that LIGHT overexpression, which activates HVEM downstream signaling, boosts CAR T cell proliferation and antitumor activity [46]. Simultaneously, Guruprasad et al. demonstrated that BTLA knockout, blocking the HVEM/BTLA axis coinhibitory signaling, promotes CAR T antitumor efficacy [47]. These findings indicate that modulating HVEM to induce costimulatory signaling and inhibit coinhibitory signaling represents a novel approach to enhancing the antitumor efficacy of CAR T cells.

The limited in vivo efficacy observed with both 4-1BB- and AVEE-mutated HVEM-CAR T cells in this study can be attributed to the subtherapeutic dosing strategy. In our previous work [9], a dose of 5 × 10^5^ CAR T cells was administered. At this dose, 4-1BB-CAR T cells demonstrated measurable anti-tumor effects compared to non-transduced T cells (Ctrl-T), while HVEM-CAR T cells showed superior efficacy, achieving complete tumor eradication in three of five mice within an orthotopic xenograft model of human renal cancer. In the current study, we deliberately selected a lower dose of 3 × 10^5^ CAR T cells per mouse to better discriminate functional differences between various CAR constructs, particularly those with signaling domain mutations. The complete lack of tumor control by AVEE-mutated CAR T cells thus likely reflects this sub-therapeutic dosing regimen, which appears insufficient to support the functional capacity of the attenuated mutant CAR. Whether this lack of efficacy under subtherapeutic conditions predisposes AVEE-mutated HVEM-CAR T cells to heightened exhaustion represents an important question for future investigation.

In summary, this study elucidates the molecular mechanisms by which HVEM CSD enhances CAR T cell antitumor efficacy, particularly against solid tumors. By recruiting a broader array of TRAFs and activating a stronger TNF signaling pathway, HVEM-CAR T cells exhibit superior metabolic reprogramming and functional persistence compared to 4-1BB-CAR T cells. The identification of the AVEE motif as a unique TRAF-binding site further highlights the innovative aspects of HVEM CSD and its potential for improving CAR T cell therapy (Fig. 6K). These findings provide important insights into the optimization of CAR T cell therapies and suggest that HVEM CSD could be a promising candidate for enhancing the clinical outcomes of CAR T cell-based treatments for solid tumors. The study’s implications extend beyond solid tumor therapies, potentially informing the development of next-generation immunotherapies that leverage enhanced TNF signaling to overcome challenges such as immune evasion and metabolic suppression in the tumor microenvironment.

## Conclusions

While 4-1BB/CD28-based CAR T cells show limited efficacy against solid tumors, HVEM-CAR T cells exhibit superior antitumor activity via enhanced TNF signaling. We demonstrate that HVEM’s intracellular domain recruits more TRAF1/2/3 and uniquely binds TRAF5 through its AVEE motif, driving stronger AKT and ERK1/2 phosphorylation and metabolic activation than 4-1BB. Disrupting AVEE abrogates TRAF binding and antitumor effects, revealing a mechanistic basis for HVEM-CAR T cell superiority in solid tumors.

## Supplementary Information

Supplementary Material 1.

## Data Availability

No datasets were generated or analysed during the current study.

## Abbreviations

2-DG: 2-Deoxy-D-glucose
AKT: Protein Kinase B
BTLA: B- and T-lymphocyte attenuator
CAR T: Chimeric antigen receptor-engineered T
CAIX: carbonic anhydrase 9
Co-IP: Coimmunoprecipitation
CSD: co-stimulatory domain
ECAR: extracellular acidification rate
ELISA: Enzyme Linked Immunosorbent Assay
E:T: effector cells: target cancer cells
FCCP: carbonyl cyanide p-trifluoromethoxy phenylhydrazone
GSEA: Gene Set Enrichment Analysis
HVEM: herpes virus entry mediator
LIGHT: lymphotoxin-like, exhibits inducible expression, and competes with HSV glycoprotein D for HVEM, a receptor expressed by T lymphocytes
LTα: lymphotoxin-α
MAPK: mitogen-activated protein kinase
MUT: AVEE motif mutation
NF-κB: nuclear factor kappa B
OCR: oxygen consumption rate
OXPHOS: oxidative phosphorylation
PBMC: peripheral blood mononuclear cells
PI3K: phosphatidylinositol 3-kinase
qRT-PCR: Real-Time Quantitative PCR
RTCA: Real Time Cellular Analysis
scFv: single chain variable fragment
SRC: spare respiratory capacity
TNFRSF: tumor necrosis factor receptor superfamily
TRAF: TNF receptor-associated factor
WT: wild type
