# Supplementary material for: HVEM costimulatory domain boosts CAR T cell efficacy against solid tumors via enhanced TRAF-mediated TNF signaling
Source: Cell Commun Signal. 2026 Jan 8;24:90. doi: 10.1186/s12964-025-02648-4 (PMC12870389; doi:10.1186/s12964-025-02648-4)
Supplement: Supplementary file 1 — Supplementary Material 1. [file 12964_2025_2648_MOESM1_ESM.docx]

**Supplementary Figures and Supplementary Figure Legends**


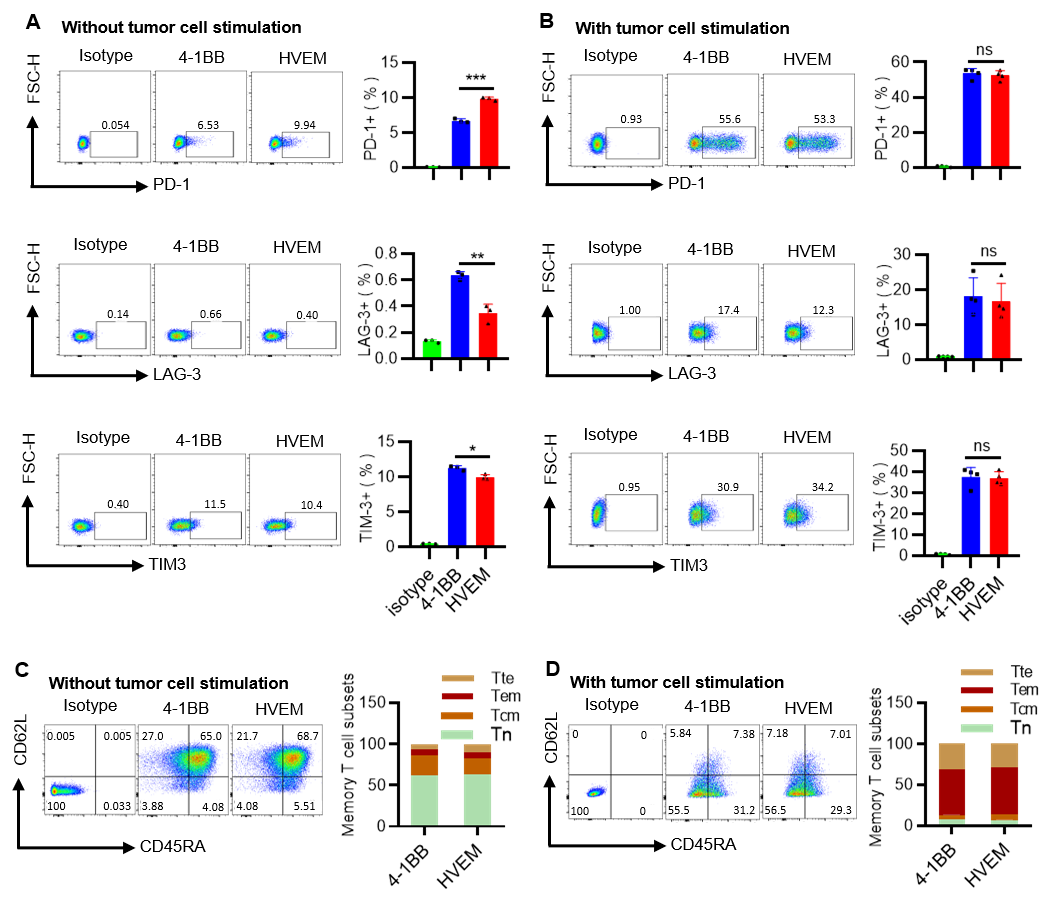


**Supplementary Figure S1. T cell exhaustion and memory phenotypes were analyzed between HVEM-CAR T cells and 4-1BB-CAR T cells.** (**A**) 4-1BB-CAR or HVEM-CAR T cells targeting CAIX antigen were generated after 10 days of expansion, then T cell exhaustion markers, PD-1, LAG-3, TIM-3, were determined using FACS. (**B**) CAIX-4-1BB-CAR or CAIX-HVEM-CAR T cells were co-cultured with OSRC-2 cancer cells for 48 hours, then levels of PD-1, LAG-3, TIM-3 in CAR-T cells were determined using FACS. (**C**) CAIX-4-1BB-CAR or CAIX-HVEM-CAR T cells were generated after 10 days of expansion, then CD62L and CD45RA were determined to illustrate the memory phenotype. naïve T cell, Tn, CD45RA+, CD62L+; central memory T cell, Tcm, CD45-, CD62L+; effector memory T cell, Tem, CD45-, CD62L-; terminal effector T cell, Tte, CD45+, CD62L-. Data are presented as mean ± SD, and analyzed via one-way ANOVA. *, *P* < 0.05; **, *P* < 0.01; ***, *P* < 0.001; ns, not significant for indicated comparison.


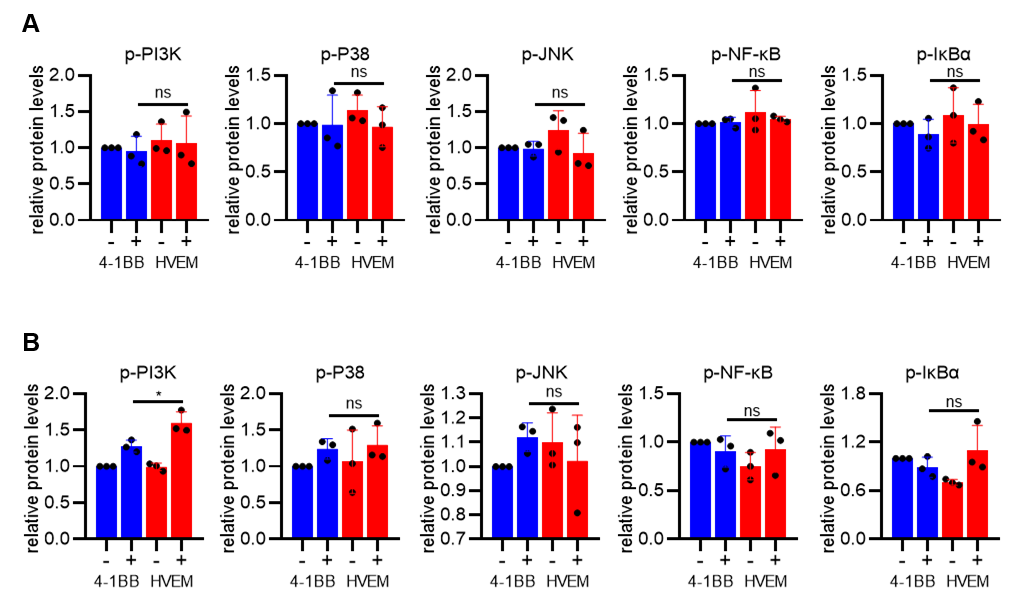


**Supplementary Figure S2. Quantification of the immunoblotting results from Figure 2C and 2E.** (**A**) Quantitative analysis of phosphorylated PI3K, P38, JNK, NF-κB，IκBα levels in Figure 2C was performed using ImageJ software, normalized to unstimulated 4-1BB-CAR T cells (4-1BB, -). (**B**) Quantitative analysis of phosphorylated PI3K, P38, JNK, NF-κB，IκBα levels in Figure 2E was performed using ImageJ software, normalized to unstimulated 4-1BB-CAR Jurkat cells (4-1BB, -). Data are presented as mean ± SD, and analyzed via one-way ANOVA. *, *P* < 0.05; ns, not significant for indicated comparison.


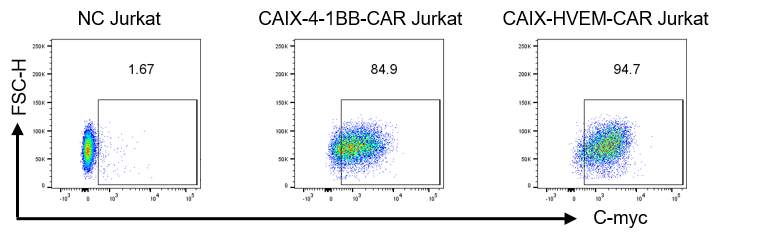


**Supplementary Figure S3. Preparations of CAIX-CAR Jurkat cells based on 4-1BB or HVEM CSD.** CAIX-CAR expression in CAR Jurkat cells were determined by flow cytometry, Jurkat cells without lentivirus transduction as control, NC Jurkat.


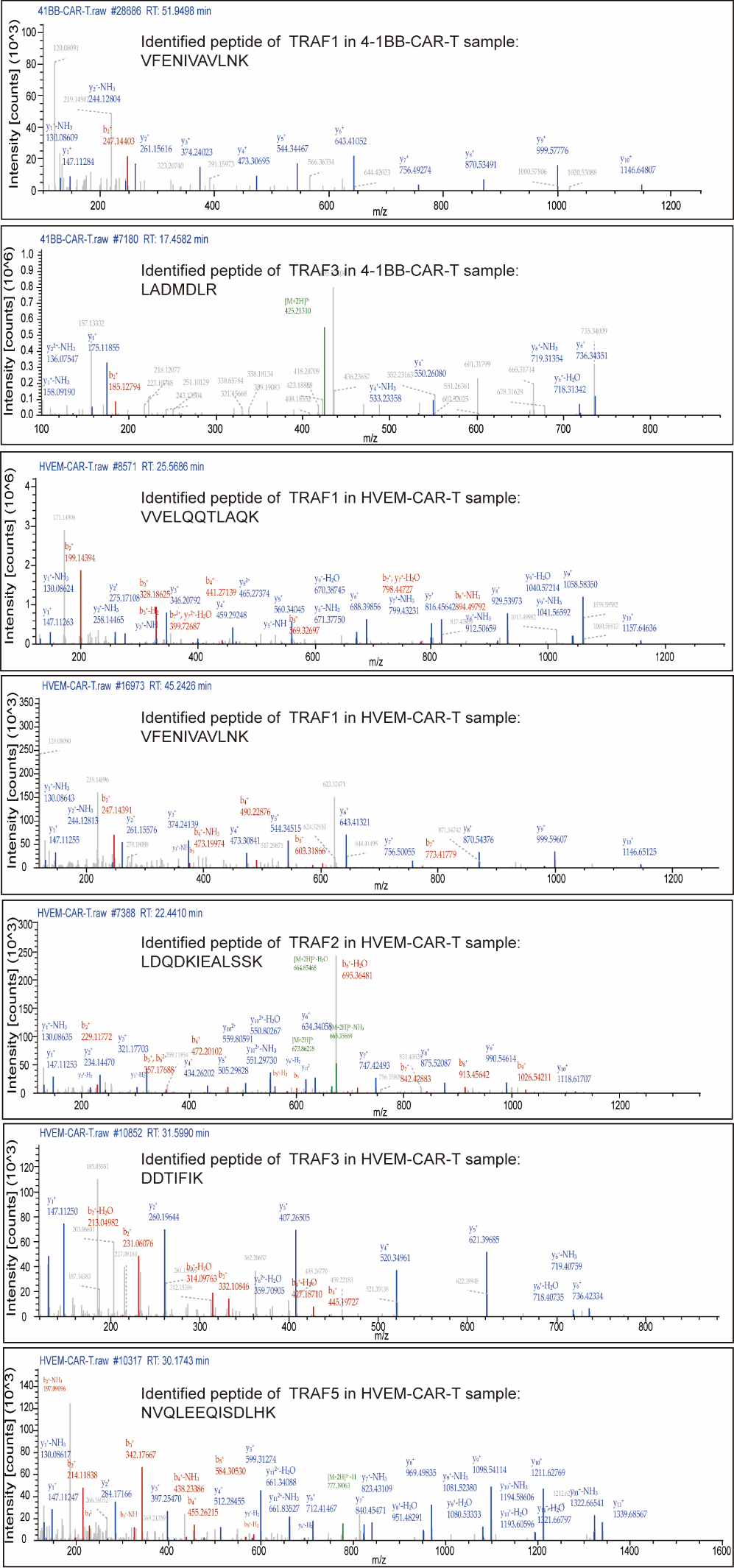


**Supplementary Figure S4. TRAFs specific peptide segments detected by mass spectrometry in CO-IP samples.** CAIX-4-1BB, or CAIX-HVEM-CAR T cells were cocultured with OSRC-2 cancer cells for 10 minutes, then CAR T cells were collected and lyzed, CARs and associated proteins were enriched by Co-IP, the sediments were analyzed using Shotgun MS. The identified TRAFs specific peptides were shown.


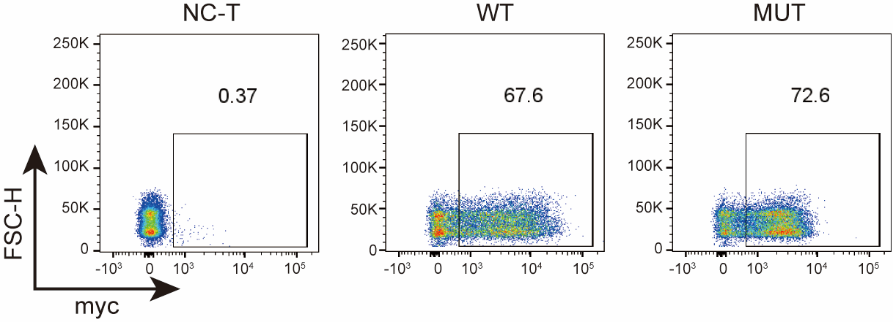


**Supplementary Figure S5. Preparation of CAIX-CAR T cells with wild-type HVEM or AVEE site mutation HVEM CSD.** Representative FACS plots of CAIX-CAR with wild-type or AVEE mutated HVEM expression on day 10 after transduction, T cells without lentivirus transduction as control, NC-T.

**Supplementary Tables**

**Supplementary Table S1. Antibodies used for Western blotting.**

| **Antibody name** | **Company** | **Catalog Number** | |
| --- | --- | --- | --- |
| Myc | Cell Signaling Technology | | 2276 |
| TRAF1 | Cell Signaling Technology | | 4715 |
| TRAF2 | Cell Signaling Technology | | 4724 |
| TRAF3 | Cell Signaling Technology | | 4729 |
| TRAF5 | Cell Signaling Technology | | 41658 |
| phospho-PI3K | Cell Signaling Technology | | 17366 |
| total-PI3K | Cell Signaling Technology | | 4249 |
| phospho-Akt (Ser473) | Cell Signaling Technology | | 4060 |
| total-Akt | Cell Signaling Technology | | 2920 |
| phospho-ERK1/2 | Cell Signaling Technology | | 4370 |
| total- ERK1/2 | Cell Signaling Technology | | 4695 |
| phospho-P38 | Cell Signaling Technology | | 4511 |
| total-P38 | Cell Signaling Technology | | 9212 |
| phospho-JNK | Cell Signaling Technology | | 4668 |
| total-JNK | Cell Signaling Technology | | 9252 |
| phospho-NF-κB | Cell Signaling Technology | | 3033 |
| total- NF-κB | Cell Signaling Technology | | 8242 |
| phospho-IκBα | Cell Signaling Technology | | 2859 |
| total-IκBα | Cell Signaling Technology | | 9242 |
| GAPDH | Proteintech | | 60004-1-Ig |

**Supplementary Table S2. Antibodies used for flow cytometry.**

| **Antibody name** | **Company** | | **clone** | **Catalog Number** |
| --- | --- | --- | --- | --- |
| PercP anti-hCD3 | | BioLegend | UCHT1 | 300428 |
| APC/Cy7 anti-hCD45 | BioLegend | | HI30 | 304014 |
| PE anti-hPD-1 | BioLegend | | EH12.2H7 | 329905 |
| PE anti-hTIGIT | BioLegend | | A15153G | 372703 |
| PE-Cy7 anti-hTIM-3 | BioLegend | | F38-2E2 | 345014 |
| PE-Cy7 anti-LAG-3 | BioLegend | | 7H2C65 | 369207 |
| PE-Cy7 anti-hCD45RA | BioLegend | | HI100 | 304126 |
| FITC anti- hCD62L | BioLegend | | DREG-56 | 104405 |
| FITC anti-hEpCAM | BioLegend | | 9C4 | 324204 |
| AF647 anti-hKi-67 | BioLegend | | Ki-67 | 350510 |
| Mouse Ab anti-C-myc | Sigma-Aldrich | | 9E10 | SAB4700447 |
| AF647 anti-mouse Ab | BioLegend | | AF6-120.1 | 116412 |

**Supplementary Table S3. shRNA sequences used for TRAFs knockdown.**

| Target gene | Target sequence |
| --- | --- |
| TRAF1 | GCCTTCTACACTGCCAAGTAT |
| TRAF2 | GTGTTCACGAGGGCATATATG |
| TRAF3 | CCAGCCTTTCTACACTGGTTA |
| TRAF5 | GAAGCTCAAGTGCATCAATTA |
| scrambled control | CAGGCCATTACTGTCTCATTG |

**Supplementary Table S4. Primer sequences used for qPCR.**

| Genes | Forward primer | Reverse primer |
| --- | --- | --- |
| *Cpt1a* | TCCAGTTGGCTTATCGTGGTG | TCCAGAGTCCGATTGATTTTTGC |
| *Fabp5* | TGAAGGAGCTAGGAGTGGGAA | TGCACCATCTGTAAAGTTGCAG |
| *Cox6b1* | CTACAAGACCGCCCCTTTTGA | GCAGAGGGACTGGTACACAC |
| *Cox7c* | GGTCCGTAGGAGCCACTATGA | GTGTCTTACTACAAGGAAGGGTG |
| *Atp5mf* | ATGGCGTCAGTTGGTGAGTG | TGAAGTCCCGCATCAAGATCC |
| *Ndufa3* | GGGGCCTCGCTGTAATTCTG | GACGGGCACTGGGTAGTTG |
| *Ndufa7* | TGCAGCTACGCTACCAGGA | GGAGGCTGAGTTCGCTTGG |
| *Ndufb9* | GTGGTGCGTCCAGAGAGAC | GGCCTTCGCCATATCCTTTTC |
| *Glut1* | ATTGGCTCCGGTATCGTCAAC | GCTCAGATAGGACATCCAGGGTA |
| *Pdk1* | GAGAGCCACTATGGAACACCA | GGAGGTCTCAACACGAGGT |
| *Pgk1* | TGGACGTTAAAGGGAAGCGG | GCTCATAAGGACTACCGACTTGG |
| *G6pd* | CGAGGCCGTCACCAAGAAC | GTAGTGGTCGATGCGGTAGA |
| *Slc16a3* | CGGCTTTGTGCTTTACGCC | GCTGAAGAGGTAGACGGAGTA |
| *Vegfa* | AGGGCAGAATCATCACGAAGT | AGGGTCTCGATTGGATGGCA |
| *β-Actin* | CATGTACGTTGCTATCCAGGC | CTCCTTAATGTCACGCACGAT |
